# Continuous adipose-derived stem cell therapy from the neonatal stage effectively reduces Duchenne muscular dystrophy symptoms in rats

**DOI:** 10.1186/s13287-025-04594-x

**Published:** 2025-08-26

**Authors:** Yuki Kihara, Masanari Ikeda, Ryo Takagi, Keiko Ishigaki, Keitaro Yamanouchi, Satoru Nagata, Masayuki Yamato

**Affiliations:** 1 Department of Pediatrics, School of Medicine, Tokyo Women’s Medical University, 8-1 Kawada-cho, Shinjuku-ku, Tokyo, 162-8666 Japan; 2 Institute of Advanced Biomedical Engineering and Science, Tokyo Women’s Medical University, 8-1 Kawada-cho, Shinjuku-ku, Tokyo, 162-8666 Japan; 3 Department of Veterinary Physiology, Graduate School of Agricultural and Life Sciences, The University of Tokyo, Bunkyo-ku, Tokyo, 113-8654 Japan

**Keywords:** Duchenne muscular dystrophy, Cell therapy, Mesenchymal stem cells, Fibrosis Suppression, Cellular senescence

## Abstract

**Background:**

The optimal timing for mesenchymal stem cell (MSC) therapy in Duchenne muscular dystrophy (DMD) remains unclear.

**Methods:**

Neonatal DMD rats received intraperitoneal adipose-derived MSCs according to three schedules: early (postnatal days 1 and 14), continuous (days 1, 14, 28, and 42), or late (days 28 and 42). Wild-type rats and untreated DMD rats served as controls. Functional and histological outcomes were assessed on day 56.

**Results:**

Continuous administration significantly attenuated the decline in grip strength across ten consecutive measurements (− 11% vs. −37% in DMD controls), and also reduced serum creatine kinase levels and diaphragmatic fibrosis (*p* < 0.05). Early or late treatment alone showed limited benefit. GFP-labelled cells were rarely detected in muscle, indicating minimal engraftment and suggesting paracrine-mediated effects. Molecular profiling showed lower CDKN2A together with higher CDKN1A, IL-10, VEGF-A and IGF-1 in the continuous group, revealing an anti-senescence, pro-regenerative profile that paralleled the functional gains.

**Conclusion:**

Early and sustained MSC administration offers superior structural and functional protection in DMD rats, highlighting the importance of treatment timing in maximizing therapeutic efficacy.

**Supplementary Information:**

The online version contains supplementary material available at 10.1186/s13287-025-04594-x.

## Background

Duchenne muscular dystrophy (DMD) is a genetic disorder characterized by progressive muscle degeneration and weakness caused by mutations in the dystrophin gene, resulting in skeletal and cardiac muscle damage [1]. Patients with advanced DMD require ventilatory support and treatment for cardiomyopathy, ultimately leading to death from respiratory infections or heart failure, giving it a poor prognosis [2]. As a treatment, corticosteroids have been extensively used [3]. Recent reports have shown the effectiveness of gene therapy [4] and exon skipping therapies [5], although they do not target all mutations. These treatments have the potential to replace steroid therapy because they address the underlying genetic causes of DMD rather than merely managing symptoms. Unlike steroids, which primarily aim to reduce inflammation and slow muscle degeneration, gene therapy and exon skipping can restore dystrophin production, thereby potentially halting or even reversing the progression of the disease.

Patients with mutations that cannot be targeted by gene therapy have to rely on steroids and other symptomatic treatments, which are not fundamental cures. However, these treatments do not halt disease progression completely [6], and long-term steroid use can lead to significant side effects [7]. In children, concerns include growth retardation in addition to adult side effects like a cushingoid appearance and osteoporosis [3, 7]. Therefore, alternative therapies are needed. Stem cell therapy, which can be administered regardless of the specific mutation, presents a promising alternative.

Stem cell therapy for DMD has been studied in various cell types, both in animal experiments [8–11] and human clinical trials [12–14], offering a treatment approach independent of genetic mutation. Animal studies have shown improvements in tissue necrosis and the emergence of dystrophin-positive muscle fibers in DMD model mice following intravenous administration of mesenchymal stem cells (MSCs) [8]. Human clinical trials have reported that intravenous administration of cardiosphere-derived cells, allogeneic heart-derived stem cells, can delay the decline in motor and cardiac functions [12]. Additionally, combined arterial and muscular injections of allogeneic MSCs have been shown to enhance motor and respiratory functions [13]. Although the exact mechanisms of stem cell action in treating DMD are not fully understood, prior research suggests that the primary mechanisms involve paracrine and endocrine effects mediated by exosomes and cytokines rather than the direct contribution of engrafted cells to skeletal muscle [15].

Despite promising reports, stem cell therapy for DMD has not been thoroughly investigated in terms of the optimal timing of administration. Clinical trials have limited stem cell therapy for patients with DMD to those of a certain body size and at a stage where motor function decline is evident, making it easier to distinguish differences from controls [12–14]. Consequently, there has been no investigation into the optimal timing for initiating treatment.

**Fig. 1:**
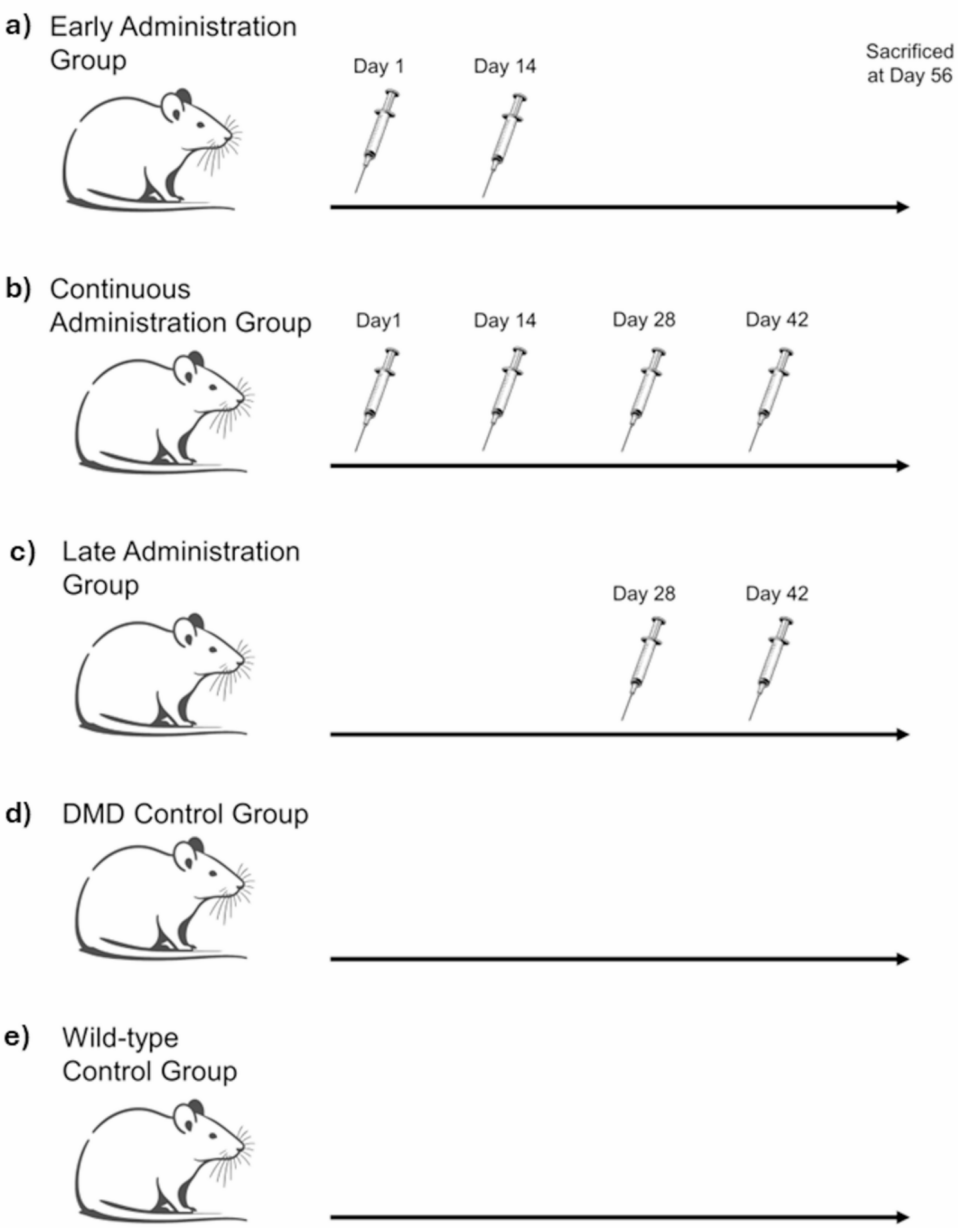
Experimental overview. The experiment included five groups: (1) early administration group; (2) continuous administration group; (3) late administration group; (4) Duchenne muscular dystrophy control group; and (5) wild-type control group, each comprising five animals. Groups 1 to 3 were injected intraperitoneally with 5.0 × 10^6^ ASCs. All animals were euthanized for analysis on postnatal Day 56 (Day 1 refers to postnatal Day 1)

Therefore, this study aimed to investigate the optimal timing for treating DMD by transplanting MSCs into neonatal DMD model rats. Administering MSCs to neonates offers potential benefits such as the integration of transplanted cells into growing muscle tissue and early suppression of inflammation responses associated with muscle degradation, which could prevent disease progression. Given that neonates have an immature immune system and are less likely to reject transplanted cells, this stage presents a unique opportunity for cell therapy. Indeed, there have been reported cases where neonates developed lung cancer due to the inhalation of cervical cancer cells from the mother during delivery [16], indicating that allogeneic transplantation can occur. Despite the potential benefits of early detection and treatment for DMD, there have been no previous studies investigating the optimal timing for initiating cell therapy in this condition. In this study, we varied the timing of MSC administration to DMD rats (Fig. 1) to determine the most effective treatment period. Our findings indicate that continuous administration starting from the neonatal period is the most effective, highlighting the importance of early intervention in DMD treatment strategies.

## Methods

### Ethics

All experiments were approved by the Ethics Committees of Tokyo Women’s Medical University and The University of Tokyo in Japan (approval number AE21-044; study title: “Investigation of Neonatal Stem Cell Transplantation in Duchenne Muscular Dystrophy Rats”, approval date: March 18, 2021). Animals were handled in accordance with guidelines from the Science Council of Japan. This study was conducted following the Animal Research: Reporting of In Vivo Experiments (ARRIVE) guidelines 2.0.

### Duchenne muscular dystrophy rats

The rats were housed at the Institute of Advanced Biomedical Engineering and Science in Tokyo, Japan, or at the University of Tokyo in separate cages with 12-hour light/dark cycles. They were provided with laboratory chow and water ad libitum.

DMD rats were generated by targeting exons 3 and 16 of the *DMD* gene using the CRISPR-Cas9 system to induce artificial out-of-frame mutations, as previously described [17]. For this study, heterozygous carrier females were mated with wild-type males to generate DMD rats for neonatal cell transplantation.

### Primary culture of adipose-derived stem cells

Adipose tissue was harvested from the abdomens of 4-week-old SD-Tg (CAG-EGFP) rats following euthanasia by CO_2_ inhalation, for primary culture, as previously described [18]. In brief, the collected fat tissue was digested using type II collagenase (Sigma-Aldrich) at 37 °C with a gentleMACS™ Octo Dissociator with Heaters (Miltenyi Biotec). The cells were then centrifuged, collected, and cultured in adipose-derived stem cell (ASC) medium (Dulbecco’s Modified Eagle Medium/F-12 GlutaMAX supplement (Gibco), 10% fetal bovine serum (FBS), and 1% penicillin-streptomycin) to promote cell proliferation.

### Characterization of adipose-derived stem cells

The characteristics of ASCs were validated through colony formation assays, flow cytometry, differentiation induction experiments and RT-qPCR. In the colony formation assay, approximately 500 cells were seeded into a 100-mm dish and cultured for seven days. After fixation with paraformaldehyde, the cells were stained with crystal violet to confirm colony formation. For flow cytometry, surface antigens of cells primarily cultured as ASCs were analyzed using antibodies against CD34, CD29, CD90, CD11, and CD45 (details of the antibodies used are listed in Supplementary Table 1). Cells were suspended in 100 µL of phosphate-buffered saline (PBS) at a concentration of 7.5 × 10^5^ cells, to which 1 µL of antibody was added. After incubation at room temperature for one hour, 1,000 µL of PBS containing 2% FBS was added for dilution, followed by centrifugation, discarding the supernatant except for 50 µL. An additional 500 µL of 2% FBS in PBS was added along with 1 µL of propidium iodide, and flow cytometry was performed using a Gallios 3 L 10 C (Beckman Coulter). Differentiation experiments were conducted as in previous studies. Osteogenic differentiation was induced by culturing ASCs in medium supplemented with 0.05 mM L-ascorbic acid 2-phosphate, 10 nM dexamethasone, and 10 mM β-glycerophosphate for 14 days, followed by staining with Alizarin Red [19]. Adipogenic differentiation was induced by culturing in MSC medium supplemented with 1 µM dexamethasone, 10 µM insulin, 0.5 mM 3-isobutyl-1-methylxanthine, and 200 µM indomethacin for 14 days, followed by Oil Red O staining [20]. To measure the expression of factors secreted by ASCs, total RNA was extracted using the RNeasy Mini Kit (QIAGEN) according to the manufacturer’s instructions. cDNA synthesis was performed using ReverTra Ace (TOYOBO). RT-qPCR was conducted using TaqMan Fast Advanced Master Mix and TaqMan probes (primers listed in Supplementary Table 3), with analysis carried out on a ViiA7 real-time PCR system (Thermo Fisher Scientific). Gene expression levels were quantified relative to glyceraldehyde-3-phosphate dehydrogenase (GAPDH), expressed as a percentage (%GAPDH).

### Coculture experiment of adipose-derived stem cells and myoblasts

A coculture experiment was conducted using a previously reported method that involves specific adhesion to Laminin-221 for primary culturing of myoblasts from green fluorescent protein (GFP)-unlabeled Wistar rats [21], which were euthanized by CO_2_ inhalation. Specifically, muscle tissue harvested from rats was dispersed using collagenase type II (Worthington Biochemical Corporation) and then seeded onto dishes coated with Laminin-221 (Nippi Inc., iMatrix-221). After two hours, the supernatant was removed, enabling the cells with a high affinity for Laminin-221 to adhere to the dishes and be selectively cultured as primary myoblasts. As a control, myoblasts were cultured and induced to differentiate without ASCs under the same conditions, in parallel with the coculture experiment.

For the coculture experiment, 5.0 × 10^4^ myoblasts were seeded onto a collagen-coated 1.7 cm² slide dish and cultured in ASC medium for 24 h. Subsequently, 5.0 × 10^3^ GFP-positive ASCs, previously cultured as described, were added to the same chamber. The culture was maintained for four days in differentiation medium (high-glucose Dulbecco’s Modified Eagle Medium, 2% horse serum, and 1% penicillin-streptomycin), followed by fixation with 4% paraformaldehyde.

After fixation, the cells were washed with PBS, permeabilized for 5 min with 0.5% Triton-X in PBS, and then washed with PBS. Blocking was performed at room temperature for 15 min using Blocking One Histo (Nacalai Tesque). Following the removal of the blocking solution with another PBS wash, primary antibodies (anti-GFP and anti-fast skeletal myosin heavy chain) were applied, and the cells were incubated overnight at 4 °C. Details of the antibodies used are listed in Supplementary Table 2.

### Stem cell transplantation

The MSCs were transplanted in three different groups: (1) early administration group; (2) continuous administration group; and (3) late administration group. Wild-type rats and untreated DMD rats were also reared during the same period and used as controls, with each group consisting of five animals.

On the first day after birth, 100 µL of saline solution containing the cells was administered intraperitoneally using a 36-gauge needle. Subsequently, 500 µL of saline solution containing the cells was injected intraperitoneally using a 30-gauge needle.

The cells were administered on postnatal days 1 and 14 for the early administration group, on postnatal days 1, 14, 28, and 42 for the continuous administration group, and on postnatal days 28 and 42 for the late administration group. This schedule was established based on the onset of dystrophic changes in the diaphragm of DMD rats, which typically occurs between postnatal days 28 and 48. Each administration involved an intraperitoneal injection of 5.0 × 10^6^ ASCs. Genotyping was avoided on Day 1 to prevent the risk of bleeding or death from tail cutting. Therefore, cells were administered to all rats in groups 1 and 2. Only the DMD rats were selected to continue the experiments. Genotyping was conducted after 1 week *via* PCR using specific primers (forward AGTTTCCATCAATAGCCATACCAAA and reverse TCTCAGTGTACAAGTGTGACGAACA), and only male rats with the mutation underwent cell transplantation.

On Day 56, grip strength and body weight were evaluated, after which rats were euthanized under deep isoflurane anesthesia by large volume blood collection from the abdominal aorta.

### Measurement of grip strength and body weight

Body weight and grip strength were measured just before euthanasia on Day 56. Grip strength was measured using a rat-specific grip strength meter, model GPM-101B (MELQUEST). The measurements were repeated ten times consecutively, and the variations and patterns from the initial to the final measurement were analyzed.

### Measurement of serum creatine kinase levels

Blood samples were collected from all rats immediately after euthanasia on Day 56 and sent to SRL Inc. for analysis. Creatine kinase (CK) levels were measured using the nicotinamide adenine dinucleotide phosphate method.

### Reverse transcription-quantitative polymerase chain reaction analysis

Tissue samples from the tibialis anterior (TA) muscle, diaphragm, liver, and lungs were collected from animals euthanized at Day 56 of age and preserved in an RNA storage reagent. The samples were stored at 4 °C for 24 h and then transferred to − 80 °C for long-term storage. For analysis, samples were thawed, and RNA was extracted using the RNeasy Fibrous Tissue Mini Kit (QIAGEN) according to the manufacturer’s instructions. cDNA synthesis was performed using ReverTra Ace (TOYOBO). RT-qPCR was conducted using TaqMan Fast Advanced Master Mix and TaqMan probes (primers listed in Supplementary Table 3), with analysis carried out on a ViiA7 real-time PCR system (Thermo Fisher Scientific). Expression levels of senescence-associated secretory phenotype (SASP) components, cyclin-dependent kinase inhibitor 1 A (CDKN1A), cyclin-dependent kinase inhibitor 2 A (CDKN2A), vascular endothelial growth factor A (VEGF-A), interleukin-10 (IL-10), insulin-like growth factor 1 (IGF-1), and tumor necrosis factor alpha (TNF-α) were quantified using the ΔΔCt method, with wild-type samples serving as the control.

### Masson’s trichrome staining

In this study, tissue samples were processed using the Masson’s trichrome staining method according to a specific protocol. The samples were first immersed in a primary staining solution (MUTO PURE CHEMICALS) for 20 min and then rinsed in running tap water for five minutes. Subsequently, they were stained with Weigert’s iron hematoxylin (FUJIFILM Wako Pure Chemical Corporation) for 20 min, followed by a color developing solution (MUTO PURE CHEMICALS) for 10 min. The samples were briefly dipped in a secondary staining solution (MUTO PURE CHEMICALS) for 10 s and rinsed under running tap water for one minute. To enhance the staining effect, the samples were treated twice briefly with a 1% acetic acid solution. They were then stained for 1 min in a 0.75% Orange G solution (MUTO PURE CHEMICALS) and treated again with a 1% acetic acid solution, repeated twice. Subsequently, the samples were stained with Masson’s staining solution B (MUTO PURE CHEMICALS) for 20 min and treated briefly with a 1% acetic acid solution, repeated twice. Following this, a 10-minute staining in a 2.5% phosphotungstic acid solution was conducted, and the samples were once more briefly treated with a 1% acetic acid solution, repeated twice. The final staining step was conducted with aniline blue for 30 min, followed by a final brief treatment with a 1% acetic acid solution, repeated twice. The samples were dehydrated through an alcohol dehydration series, cleared in xylene, and mounted in a suitable medium for analysis.

Stained tissue samples were examined under a fluorescence microscope, BZ-X810 (Keyence), and the area of the blue-stained fibrotic regions was measured using the image analysis software BZ-H4C (Keyence). The percentage of the fibrotic area was calculated by dividing it by the total area.

### Immunofluorescence staining

The harvested TA muscle and diaphragm were fixed to cork using tragacanth gum and snap-frozen in isopentane cooled with liquid nitrogen. The tissues were sectioned into 6-µm thick cryo-tissue sections using a cryostat and fixed with acetone for 5 min before immunostaining. After washing with PBS, the sections were permeabilized with 0.5% Triton-X in PBS for five minutes and washed again before incubating at room temperature for 60 min with Blocking One Histo. Subsequently, the sections were washed with PBS to remove the blocking solution, and primary antibodies (anti-GFP, anti-dystrophin, anti-MYH3, and anti-laminin listed in Supplementary Table 2) were applied and incubated overnight at 4 °C.

After incubation with secondary antibodies and Hoechst for 45 min at room temperature, the sections were washed with PBS and mounted. The stained tissue samples were examined under a fluorescence microscope, BZ-X810, and the area of embryonic myosin heavy chain-positive fibers was quantified using the image analysis software BZ-H4C. The percentage of regenerative fibers was calculated by dividing the positive area by the total area.

### In situ hybridization

ISH was conducted using an ISH Reagent Kit (Genostaff) according to the manufacturer’s instructions. Tissue sections were fixed in 10% neutral-buffered formalin for 30 min at 37 °C, washed in distilled water, treated with 0.2% hydrochloric acid for 10 min at 37 °C, and then placed in a Coplin jar with 1× G-Wash (Genostaff; #GW-01), equivalent to 1× SSC. Hybridization was performed with 250 ng/mL of probes in G-Hybo-L (Genostaff; #RPD-02) for 16 h at 60 °C. Following hybridization, the sections were washed three times with 50% formamide in 2× G-Wash for 30 min at 50 °C and five times in Tris-Buffered Saline with Tween (TBST; 0.1% Tween20 in TBS) at room temperature. Subsequently, the sections were treated with 1× G-Block (Genostaff; GB-01) for 15 min at room temperature and then incubated with anti-DIG AP conjugate (Roche; #11093274910) diluted 1:2000 with G-Block (1/50) in TBST for one hour at room temperature. The sections were washed twice in TBST and incubated in a solution containing 100 mM NaCl, 50 mM MgCl_2_, 0.1% Tween20, and 100mM Tris-hydrochloric acid (pH 9.5). Color reactions were developed using NBT/BCIP Solution (NBT; #N6876, BCIP; #B8503, Sigma-Aldrich) and washed in PBS. The sections were counterstained with Kernechtrot Stain Solution (Muto; #40,872) and mounted with G-Mount (Genostaff; #GM-01), followed by Malinol (Muto; #20,093). The diaphragm of GFP rats was used as a positive control.

Images were captured using a NanoZoomer S210 Digital slide scanner: C13239-01 (Hamamatsu Photonics) and NDP.view2 Plus Viewing software: U12388-02 (Hamamatsu Photonics).

### Statistical analysis

Group differences among the five treatment arms were examined by one-way analysis of variance (ANOVA) followed by Dunnett’s post-hoc multiple-comparison test (GraphPad Prism v8). Data are expressed as mean ± standard error of the mean (SEM), and *P* < 0.05 was considered significant.

Pair-wise associations among functional indices (body weight, rate of grip-strength decline), biochemical markers (serum creatine kinase), histological parameters (fibrotic area, eMHC-positive area) and molecular transcripts (TGF-β1, IL-6, IL-1β, CTGF, MMP-2, TNF-α, CDKN2A, CDKN1A, VEGF-A, IL-10 and IGF-1) were assessed in all animals (*n* = 25) using two-tailed Spearman rank-correlation coefficients (ρ). Resulting ρ values and exact *P*-values were adjusted for multiple testing with the Holm–Šidák method; adjusted *P* < 0.05 was accepted as statistically significant. The complete ρ matrix was visualised as a diverging red–blue heat-map (scale − 1 to + 1), and cells meeting the significance threshold are denoted by an asterisk (*). Post-hoc power analyses were conducted using G*Power 3.1 based on the observed effect sizes (η²) obtained from one-way ANOVA. The calculated power (1 − β) was 1.000 for grip strength decline (η² = 0.505), 1.000 for eMHC-positive fiber ratio in the diaphragm (η² = 0.475), 0.971 for CDKN2A expression (η² = 0.162), and 1.000 for CDKN1A expression (η² = 0.306), indicating that the sample size (*n* = 5 per group) provided sufficient statistical power for these key endpoints.

## Results

### Characteristics of the transplanted adipose-derived stem cells

**Fig. 2:**
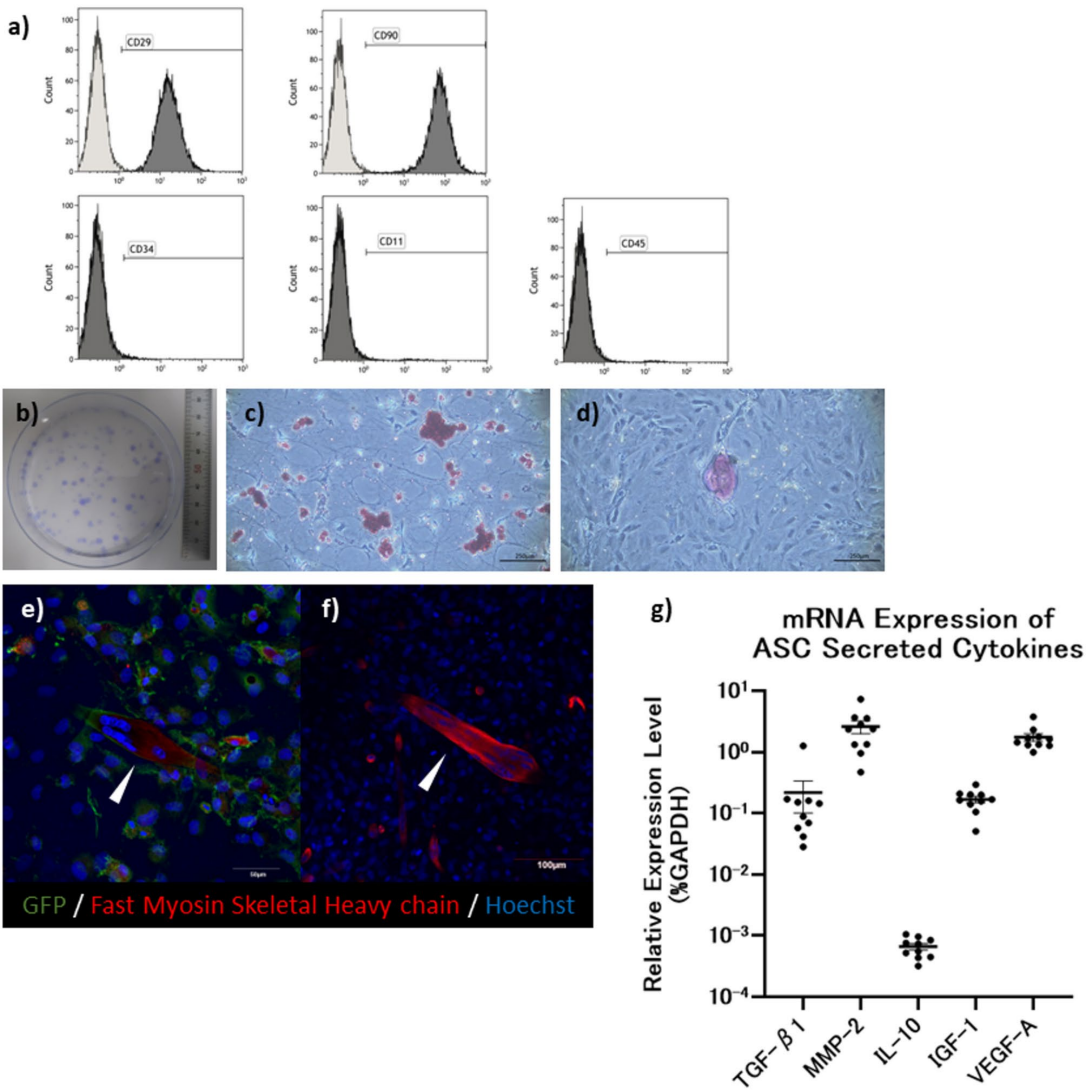
Characterization of adipose-derived stem cells. (**a**) Adipose-derived stem cells (ASCs) were analyzed using flow cytometry. The results showed strong expression of CD29 and CD90, typical of mesenchymal stem cells, while levels of hematopoietic markers CD34, CD11b/c, and CD45 were similar to the control. (**b**) Confirmation of the colony-forming ability of cultured ASCs. (**c**) Adipogenic differentiation of ASCs in adipogenic induction medium confirmed by Oil Red O staining. (**d**) Osteogenic differentiation of ASCs in osteogenic induction medium confirmed by Alizarin Red staining. (**e**) Induction of differentiation in coculture of green fluorescent protein (GFP)-positive ASCs with GFP-negative myoblasts resulting in GFP-positive myotubes, suggesting potential cell fusion between ASCs and myoblasts leading to the expression of exogenous proteins. (**f**) Differentiation of GFP-negative myoblasts without coculture with GFP-positive ASCs. As expected in this negative control, no GFP-positive myotubes were observed, indicating that GFP expression in (**e**) did not originate from the myoblasts themselves. (**g**) RT-qPCR analysis confirmed detectable mRNA expression of TGF-β1, MMP-2, IL-10, IGF-1, and VEGF-A in ASC. Expression levels were normalized to GAPDH and are presented as relative values (%GAPDH). Among these cytokines, MMP-2 and VEGF-A exhibited significantly higher expression compared to TGF-β1, IL-10, and IGF-1 (*p* < 0.05)

ASCs were primarily cultured from adipose tissue of transgenic rats SD-Tg (CAG-GFP), which express an enhanced GFP (eGFP) and analyzed *via* flow cytometry. The cells strongly expressed CD29 and CD90, which are characteristic markers of MSCs [22]. In contrast, the expression levels of hematopoietic stem cell markers such as CD34, CD11b/c, and CD45 were equivalent to those of the control group, represented by the white peaks in the flow cytometry analysis (Fig. 2a).

The cultured cells demonstrated colony-forming ability, as confirmed by crystal violet staining (Fig. 2b), and possessed the capacity to differentiate into adipocytes (Fig. 2c) and osteoblasts (Fig. 2d).

In coculture experiments, where GFP-negative myoblasts were cultured in a proliferation medium for 24 h, followed by the addition of GFP-positive ASCs and switching to a differentiation medium, GFP-positive myotubes were observed (Fig. 2e). The emergence of GFP-positive myotubes did not occur when GFP-negative myoblasts were induced to differentiate alone (Fig. 2f), indicating that the coculturing led to cell fusion between ASCs and myoblasts, resulting in the formation of myotubes expressing exogenous GFP protein. This confirms the potential of ASC fusion to express exogenous proteins.

RT-qPCR analysis showed that the mRNA expression levels of matrix metalloproteinase-2 (MMP-2) and VEGF-A were significantly higher than those of TGF-β1, IL-10, and IGF-1 in ASC (Fig. 2g). While these data provide insight into the transcriptional profile of ASC, they do not necessarily reflect functional activity.

### Transplantation of adipose-derived stem cells in neonatal Duchenne muscular dystrophy rats

ASC transplantation was intraperitoneally performed in neonatal DMD rats at a dose of 5.0 × 10^6^ cells. The rats were divided into three groups: (1) early administration group; (2) continuous administration group; and (3) late administration group, each consisting of five rats. No mortality or allergic reactions were observed in any group up to the 56-day endpoint.

**Fig. 3:**
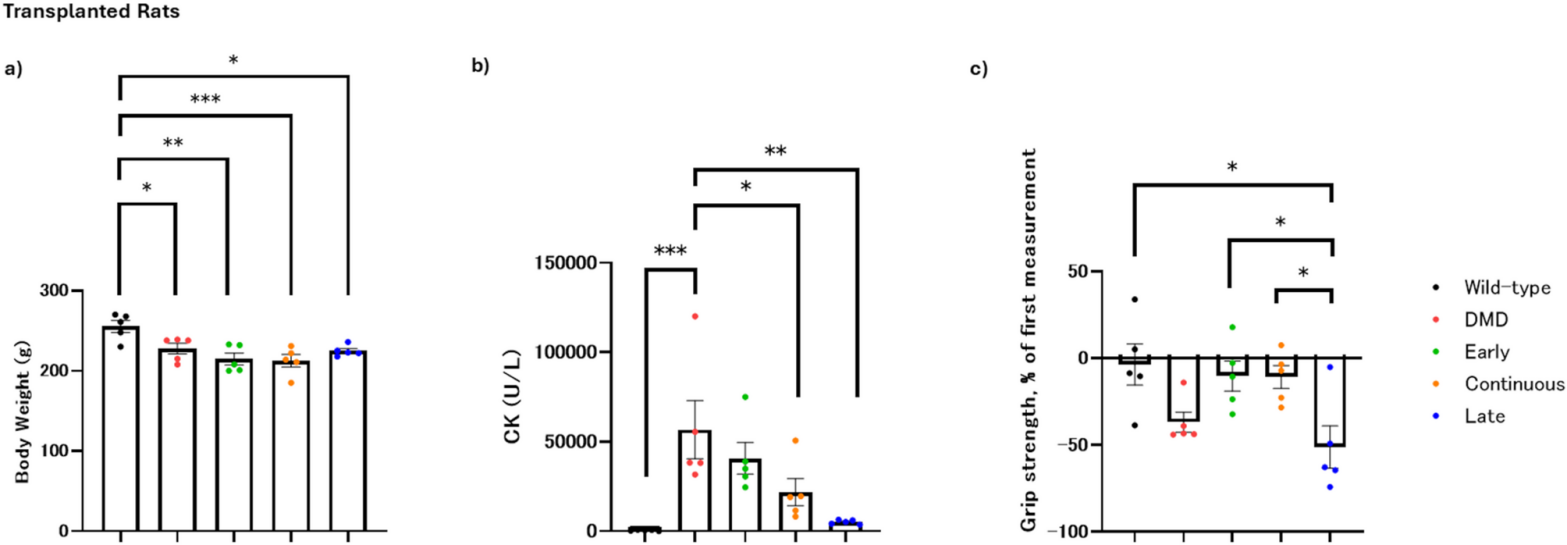
Comparison of body weight, creatine kinase, and grip strength in transplanted rats. (**a**) Compared to wild-type rats, all transplanted groups and Duchenne muscular dystrophy (DMD) rats exhibited significantly lower body weights. Cell transplantation did not significantly mitigate weight loss. (**b**) Creatine kinase (CK) levels decreased in all transplanted groups, with the continuous and late groups showing a significant reduction. (**c**) The grip strength changes from the first to the tenth measurement in a series of 10 consecutive tests was assessed. No statistically significant differences were observed compared to the DMD group, but the continuous and early groups demonstrated a significantly lower rate of grip strength decline compared to the late group, indicating improved endurance. Each dot represents an individual rat. Color-coded dots indicate groups as follows: red = wild-type control group, blue = DMD control group, orange = early administration group, green = continuous administration group, and purple = late administration group

At Day 56, the body weight of all DMD groups was significantly lower compared to the wild-type control. The differences were significant for the untreated DMD control (*p* = 0.027), early administration (*p* = 0.0013), continuous administration (*p* = 0.00070), and late administration (*p* = 0.014) groups (Fig. 3a).

At Day 56, both the continuous (*p* = 0.040) and late administration (*p* = 0.0022) groups had significantly lower serum CK levels compared to the untreated DMD control group. However, the early administration group did not exhibit a statistically significant reduction in CK levels compared to the untreated DMD control group (*p* = 0.54) (Fig. 3b).

The rate of decline in grip strength from the first to the tenth measurement was used as a parameter to assess muscle endurance. The wild-type control group exhibited the lowest decline in grip strength (− 3.8% ± 12%), followed by the early administration (− 10% ± 8.7%) and continuous administration (− 11% ± 6.5%) groups. In contrast, the late administration group experienced a substantial decline (− 51% ± 12%), similar to the untreated DMD group (− 37% ± 5.8%) (Fig. 3c). Statistical analysis using one-way analysis of variance confirmed significant differences among the groups (F (4, 20) = 4.7, *p* = 0.0074), and the sequential grip strength plots indicated that groups with higher decline rates experienced a progressive decrease in grip strength (Supplementary Fig. 1). Although no treatment group showed statistically significant differences compared to the DMD group, both the early administration (*p* = 0.041) and continuous administration (*p* = 0.044) groups demonstrated significantly lower rates of decline in grip strength compared to the late administration group (Fig. 3c).

### Histological examination of fibrosis and muscle regeneration

**Fig. 4:**
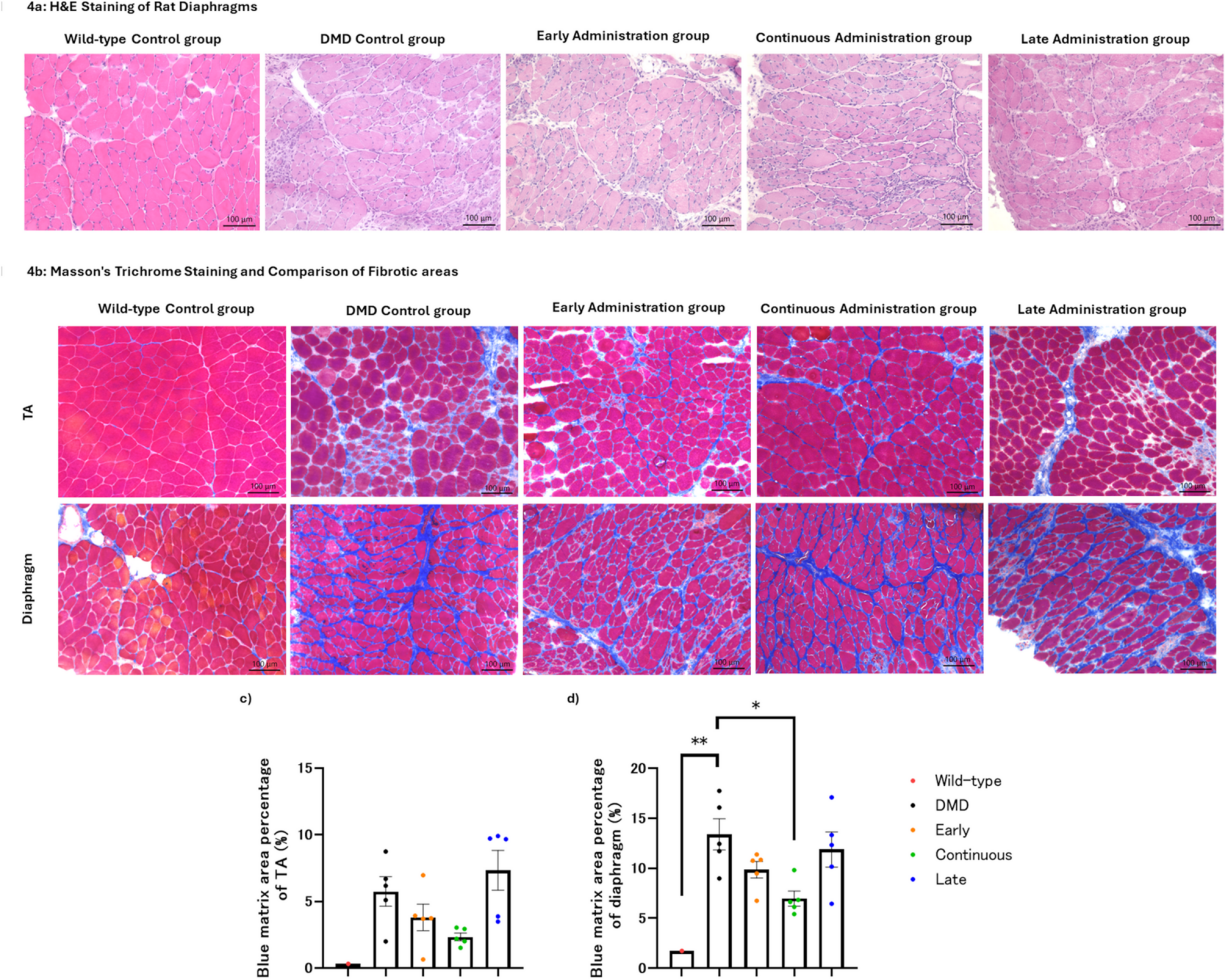
Histological analysis of muscle fibrosis in DMD rats. (**a**) Representative hematoxylin and eosin (H&E) staining images of diaphragm sections from each group: wild type control, DMD control, early administration, continuous administration, and late administration groups. All DMD-derived groups exhibited central nuclei and variation in muscle fiber size, which are characteristic features of DMD pathology. (**b**) Representative Masson’s trichrome staining images of tibialis anterior (TA) and diaphragm muscles. Fibrotic areas are stained blue. The wild type group showed minimal fibrosis, whereas transplanted groups exhibited reduced fibrotic deposition compared to the DMD control group. The continuous administration group had the smallest fibrotic area among the transplanted groups. (**c**, **d**) Quantitative analysis of fibrotic area in TA (**c**) and diaphragm (**d**) muscles. In TA, the continuous administration group had the lowest mean fibrotic area, though not statistically significant. In the diaphragm, both the wild type and continuous groups showed significantly reduced fibrotic areas compared to the DMD control group. Each dot represents an individual rat. Color coded dots indicate groups as follows: red = wild type control group, blue = DMD control group, orange = early administration group, green = continuous administration group, and purple = late administration group

Histological analysis was conducted on the TA muscle and diaphragm of rats sacrificed on Day 56. Pathological changes characteristic of DMD, such as central nuclei and variations in fiber size, were observed in all cell therapy groups. There were no significant microscopic changes compared to the untreated DMD control group (Fig. 4a).

Masson’s trichrome staining revealed that the area of fibrosis was relatively less extensive in the continuous administration group under high magnification (Fig. 4b). Image analysis was used to distinguish the blue-stained fibrotic areas from the rest, and the ratio of the fibrotic area to the total area was calculated (Supplementary Fig. 2). In both the TA muscle (Fig. 4c) and diaphragm (Fig. 4d), the continuous treatment group exhibited the smallest area of fibrosis, with the diaphragm analysis showing a statistically significant smaller area compared to the DMD control group (*p* = 0.011).

**Fig. 5:**
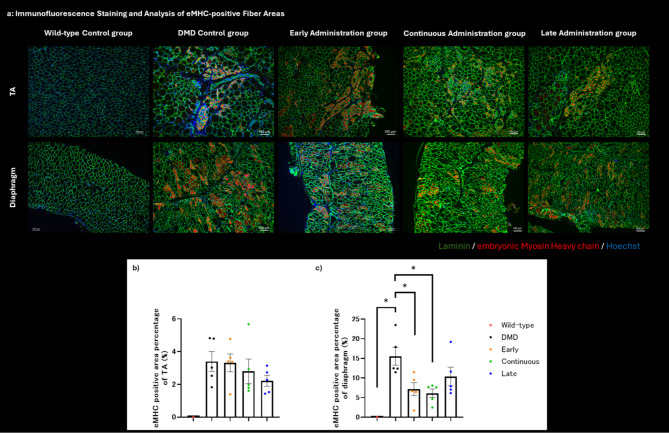
Immunofluorescence staining and Analysis of eMHC-positive fiber areas. (**a**) The figure shows magnified images of areas with the highest number of embryonic myosin heavy chain (eMHC)-positive fibers in each group. eMHC is a protein expressed in immature fibers, indicating regenerating fibers. A higher number of positive fibers suggest more active necrosis and regeneration. No positive fibers were observed in the wild-type group. Compared to the Duchenne muscular dystrophy control group, all cell-transplanted groups had fewer eMHC-positive fibers, suggesting that cell transplantation may reduce necrosis and regeneration. (**b**) The comparison of eMHC-positive fiber areas in the TA muscle showed that the late administration group exhibited the smallest positive area, although no statistically significant difference was found. (**c**) The comparison of eMHC-positive fiber areas in the diaphragm showed that the areas were significantly smaller in the wild-type, early administration, and continuous administration groups, indicating statistically significant differences. The TA muscle initially had fewer positive fibers, suggesting that data from the diaphragm more accurately reflects the therapeutic effects. Early treatment with adipose-derived stem cells (ASCs) may efficiently suppress necrosis regeneration. Each dot represents an individual rat. Color coded dots indicate groups as follows: red = wild type control group, blue = DMD control group, orange = early administration group, green = continuous administration group, and purple = late administration group

In DMD, muscle fiber necrosis and regeneration are characteristic pathological findings. Embryonic myosin heavy chain (eMHC) is known to be expressed in newly formed muscle fibers as a result of necrosis and regeneration [22]. We observed a lower presence of eMHC in all cell therapy groups compared to the DMD control group (Fig. 5a). To quantify necrosis and regeneration, the proportion of areas positive for eMHC was examined using image processing (Supplementary Fig. 3). In the TA muscle, the late administration group showed a decreasing trend, although no statistically significant difference was observed (Fig. 5b). In the diaphragm, the continuous administration group exhibited the lowest levels, with both the early administration (*p* = 0.025) and continuous administration (*p* = 0.012) groups showing statistically significant lower levels compared to the DMD control group (Fig. 5c).

### Detection of enhanced green fluorescent protein and dystrophin

To assess the engraftment and production of normal dystrophin by transplanted cells, immunofluorescence staining was conducted using anti-GFP and anti-dystrophin antibodies in the TA muscle and diaphragm. Despite examining diaphragm and TA muscle tissues from all rats, no fibers positive for eGFP or dystrophin were observed (data not shown). Furthermore, reverse transcription-quantitative polymerase chain reaction (RT-qPCR) analysis was conducted to detect eGFP mRNA in various organs, including the TA, diaphragm, lungs, and duodenum. Although eGFP mRNA was detected in some organs, there was no trend related to the timing of administration, and the expression levels were very low compared to those in GFP rats, which express eGFP in all cells (Table 1).

**Table 1:** Reverse transcription-quantitative polymerase chain reaction (RT-qPCR) analysis of enhanced green fluorescent protein (eGFP) mRNA expression in various organs

Administration Method	ID Number	Organ	Expression Level (ΔΔCt Method)
Early	D-2	TA	1.49 × 10-5
	D-6	Lung	1.34 × 10-5
		duodenum	5.88 × 10-6
Continuous	E-2	duodenum	2.40 × 10-6
	E-4	TA	7.58 × 10-4
		Diaphragm	1.62 × 10-3
Late	G-3	duodenum	5.48 × 10-6
	G-6	TA	1.35 × 10-6
	G-7	Diaphragm	4.57 × 10-6

mRNA was extracted from TA muscle, diaphragm, lung, liver, and duodenum, and RT-qPCR was performed to detect eGFP mRNA expression. eGFP mRNA expression was detected in a limited number of organs, with very low expression levels compared to rats expressing eGFP systemically. Immunofluorescence staining did not reveal eGFP-positive fibers, suggesting that engrafted cells were scarce. The formation of normal muscle cells through cell fusion was minimal and likely insufficient to contribute significantly to symptom improvement. In the continuous administration group, in situ hybridization (ISH) was performed on the diaphragm of rats positive for eGFP mRNA. However, no eGFP-positive fibers were detected (Supplementary Fig. 4).

### Quantification of senescence-associated markers, cytokines, and regenerative growth factors

**Fig. 6:**
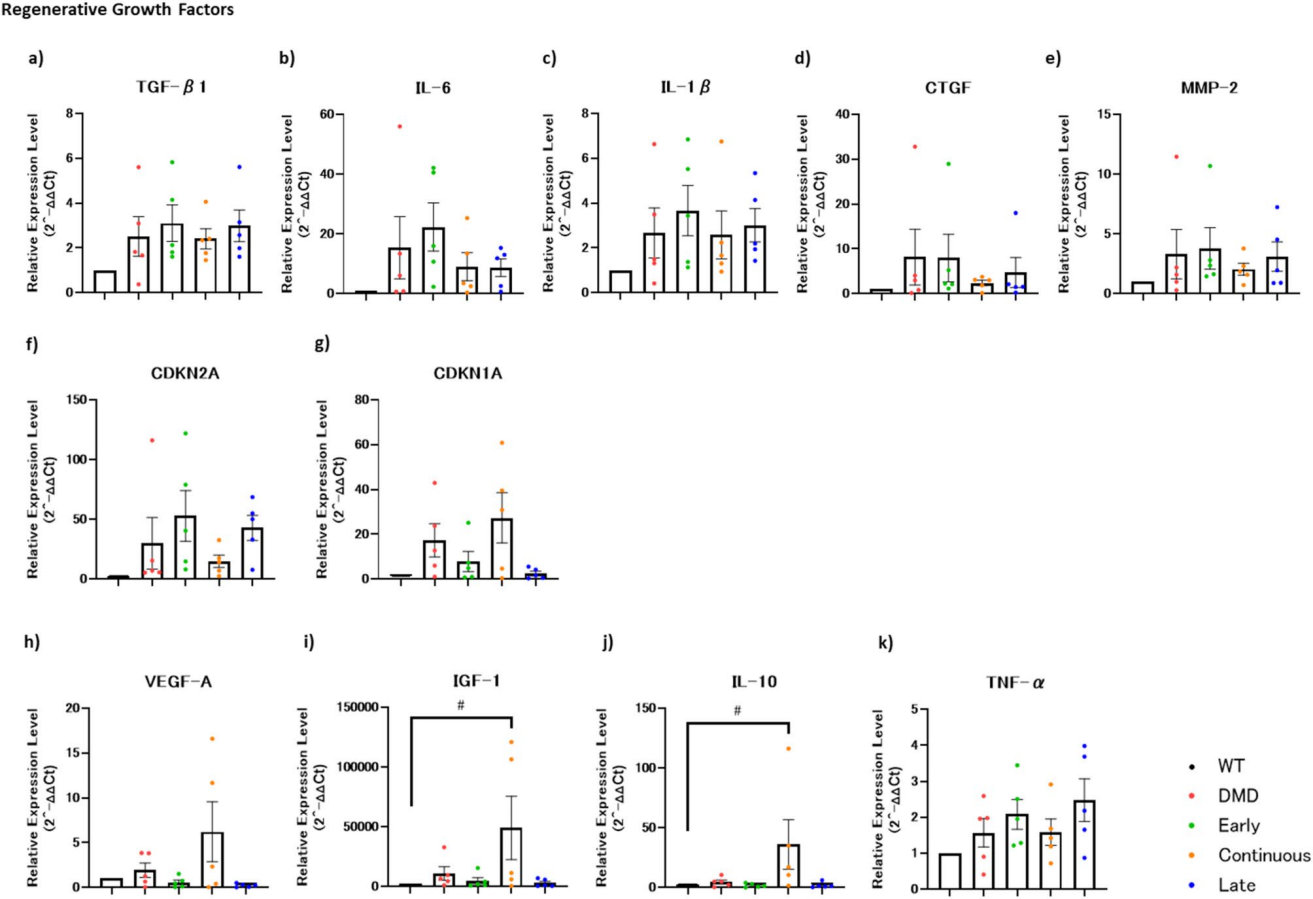
Quantification of senescence-associated secretory phenotype markers, cytokines, and regenerative growth factors. (**a**-**e**) mRNA was extracted from the diaphragm, and the expression levels of previously reported SASP factors and inflammatory cytokines were analyzed using RT-qPCR with the ΔΔCt method, normalized to the expression levels in the wild-type. No statistically significant differences were found in any of the SASP factors. In all factors, the expression levels tended to be lower in the continuous group compared to the DMD group. In the early group, the expression levels tended to be higher than in the DMD group. (**f**, **g**) Expression levels of CDKN2A and CDKN1A to further assess cellular senescence. The continuous group showed lower CDKN2A and higher CDKN1A expression, consistent with reduced senescence and enhanced differentiation. (**h**-**k**) Expression of regeneration- and inflammation-related genes. VEGF-A, IGF-1, and IL-10 were highest in the continuous group, with IGF-1 and IL-10 showing significant increases. TNF-α tended to be lower in the continuous group. Each dot represents an individual rat. Color coded dots indicate groups as follows: red = wild type control group, blue = DMD control group, orange = early administration group, green = continuous administration group, and purple = late administration group

Senescent cells cease their cell cycle and release various cytokines known as the SASP factors [23, 24]. These levels have been reported to increase in DMD rats [25]. In this study, the mRNA expression levels in muscle tissues were analyzed using RT-qPCR for transforming growth factor beta 1 (TGF-β1), interleukin-6 (IL-6), interleukin-1 beta (IL-1β), connective tissue growth factor (CTGF), and, MMP-2. To further evaluate cellular senescence, inflammation, and regeneration, additional genes were analyzed, including CDKN1A and CDKN2A as markers of senescence, VEGF-A and IGF-1 as indicators of regenerative capacity, and IL-10 and TNF-α as anti-inflammatory cytokines. Although no statistically significant differences were observed across all SASP factors, there was a trend toward lower levels in the continuous administration group (Fig. 6a-e). To investigate mechanisms related to senescence and regeneration, expression levels of CDKN2A and CDKN1A were analyzed. The continuous group showed low CDKN2A and high CDKN1A expression, suggesting suppression of irreversible senescence and promotion of myoblast differentiation. In contrast, the early and late groups exhibited the opposite pattern, indicating limited therapeutic effects and increased cellular senescence (Fig. 6f, g). This expression pattern was consistent with the group-specific SASP secretion profiles. Among the genes associated with regeneration and inflammation, VEGF-A, IGF-1, and IL-10 expression levels were highest in the continuous administration group. Notably, IGF-1 and IL-10 showed statistically significant increases compared to the other groups. In contrast, TNF-α expression tended to be lower in the continuous group than in the early and late administration groups. (Fig. 6h-k)

**Fig. 7:**
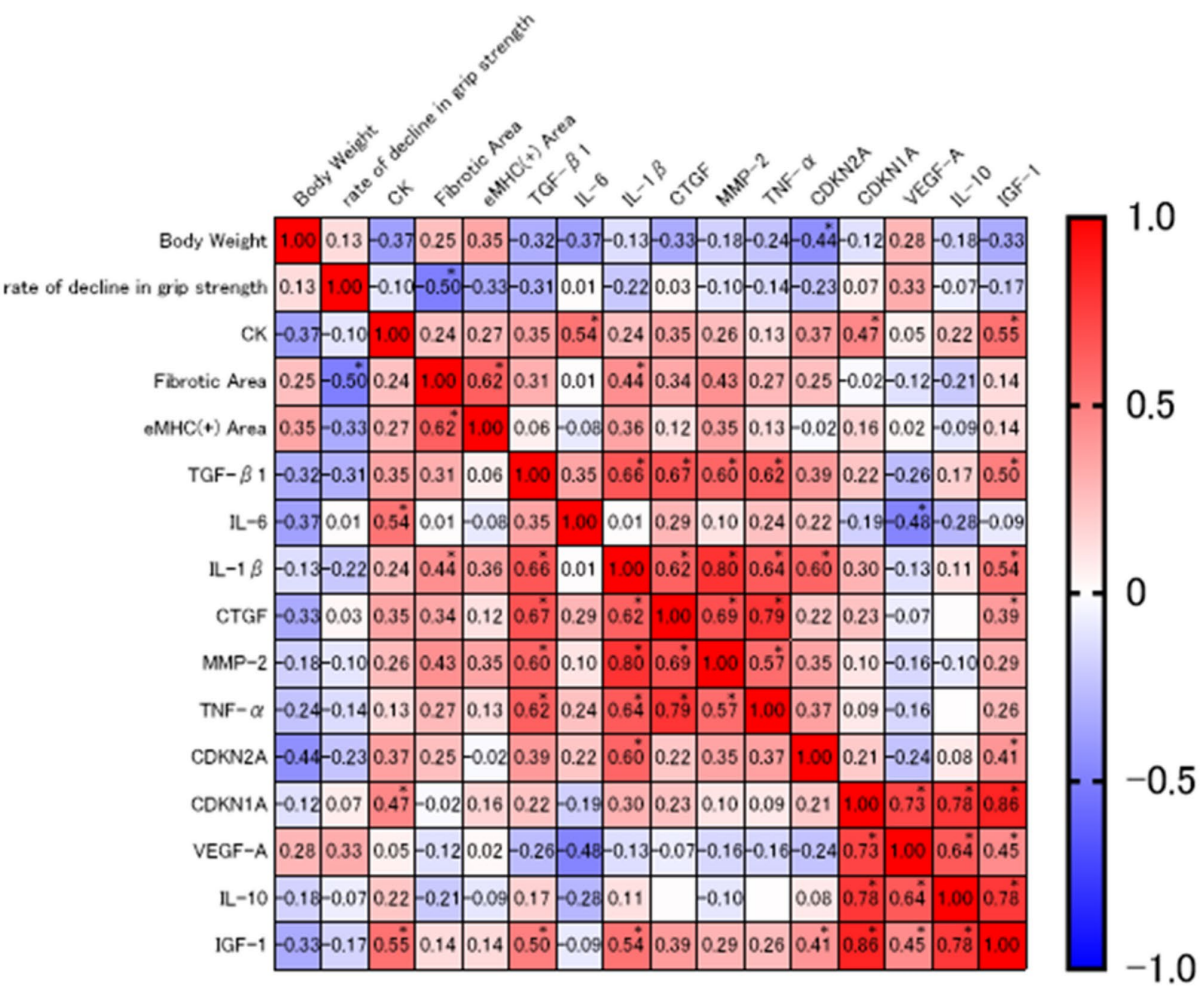
Spearman correlation heat-map of functional, histological and molecular variables. The matrix shows pair-wise Spearman rank-correlation coefficients (ρ) for body-weight, grip-strength decline, serum creatine kinase (CK), fibrotic area, eMHC-positive area and the indicated mRNA transcripts. Colors indicate the magnitude and sign of the correlation (red = positive, blue = negative; scale − 1 to + 1). Cells marked with an asterisk remain significant after Holm–Šidák adjustment of *p*-values (*p* < 0.05). Two major clusters are evident: (1) pro-fibrotic/inflammatory genes (TGF-β1, IL-1β, CTGF, MMP-2, TNF-α) and (2) regenerative/anti-inflammatory factors (CDKN1A, VEGF-A, IGF-1, IL-10). Grip strength correlates negatively with fibrosis and positively with regenerative markers, supporting an anti-fibrotic, pro-regenerative effect of continuous ASC treatment

To explore how these molecular changes relate to function, we generated a Spearman heat-map of 15 variables encompassing functional, histological and transcript data (Fig. 7). Two clusters emerged. Cluster 1 grouped the pro-fibrotic/inflammatory genes TGF-β1, IL-1β, CTGF, MMP-2 and TNF-α—the same factors that trended downward with continuous treatment. Cluster 2 linked the regenerative/anti-inflammatory set CDKN1A, VEGF-A, IGF-1 and IL-10 that was up-regulated in the continuous group. Grip-strength decline correlated inversely with diaphragm fibrosis (ρ = −0.50, *p* = 0.020). Thus, continuous ASC administration not only shifts individual gene expression but also remodels the broader molecular network toward an anti-fibrotic, pro-regenerative state that parallels functional improvement.

## Discussion

This study aimed to investigate the most effective methods of stem cell therapy for patients with DMD as it progressed toward human clinical trials. Additionally, the experiments were designed based on the hypothesis that stem cell transplantation in neonates, whose immune systems are not fully developed, might lead to donor cell engraftment.

In vitro experiments confirmed that ASCs could fuse with myoblasts and form myotubes, expressing the exogenous protein GFP, suggesting the potential for exogenous protein expression through cell fusion. There are several reports of MSC engraftment following intramuscular injection [11, 26]. In contrast, studies using intravenous or intra-arterial delivery show limited success, either detecting only a few dystrophin-positive fibers [27, 28] or reporting PCR findings without histological confirmation [15]. Due to the technical difficulty of intravenous injections in neonatal rats, this study used intraperitoneal injection as an alternative. Possibly as a result of using this route, in vivo experiments did not show GFP-positive fibers in tissue immunofluorescence staining or ISH. Only small amounts of eGFP mRNA were detected by RT-qPCR, with no clear trends based on the administration method. The undetectable levels in immunostaining and the highest expression level in the administration model (at most 1/1000 of that in GFP rats, which express eGFP in all cells) suggest minimal engraftment, if any, and an unlikely contribution to muscle recovery. Originally, MSCs, including ASCs, are known to migrate to inflamed sites [29]. Therefore, in muscular dystrophy, where inflammation is constantly present in muscle tissues, it was hypothesized that even venous or intraperitoneal administration might allow ASCs to reach necrotic muscle tissue due to enhanced vascular permeability caused by inflammation. However, actual engraftment did not occur. These results suggest that the observed mitigating effects on phenotype, even with neonatal administrations, are primarily due to paracrine or endocrine effects mediated by substances such as exosomes.

To further validate these phenotypic improvements in a clinically relevant context, we examined the effects of MSC administration on established biomarkers and functional outcomes in DMD. Serum CK, a muscle-derived enzyme, is frequently used as a biomarker in clinical settings [30]. One issue with CK is that it does not solely reflect disease progression, as it can fluctuate easily with rest or exercise [31] and may also show low values reflecting muscle loss in later stages [30]. Despite many parameters being most suppressed in the continuous administration group, CK levels were most inhibited in the late administration group. This phenomenon is often observed in clinical practice: improvements in motor functions with treatment can lead to increased physical activity, causing CK levels to rise paradoxically, leading to contradictory outcomes. The gold standard for evaluating therapeutic effectiveness is a motor function assessment. In terms of grip strength, a key measure, the continuous administration group showed better results, suggesting that continuous administration from an early stage may have a beneficial impact on treatment outcomes.

In the histological evaluation, hematoxylin and eosin staining did not show effects significant enough to eliminate the characteristic features of DMD, but the continuous administration group notably exhibited suppression of necrosis, regeneration, and fibrosis. In the TA muscle, there were no significant differences in fibrosis or the eMHC fiber positive area. This is consistent with a previous study [17], suggesting that the TA muscle undergoes fewer changes compared to the diaphragm. The experimental setup did not lead to changes in body weight in any administration method, but it did suppress CK elevation, endurance decline, SASP secretion, fibrosis, and necrosis. Continuous administration from the neonatal period was found to be the most effective in suppressing fibrosis, necrosis, and regeneration and maintaining muscle endurance. Additionally, administering stem cells only in the early period resulted in higher levels of inflammatory cytokines compared to the untreated DMD group, indicating that this approach should be avoided. This indicates that while initial treatment effects can improve motor function and activity levels, discontinuing treatment during the progression phase may worsen outcomes by creating conditions more conducive to muscle breakdown.

To elucidate the underlying molecular mechanisms responsible for these observed phenotypic and functional outcomes, we next focused on the immunological and paracrine signatures induced by ASC treatment. In mdx mice, a DMD model, systemic MSC administration has been shown to increase miR-499-5p expression, which is typically reduced in DMD. This upregulation reduces muscle fibrosis by downregulating TGF-β receptors and inhibiting SMAD2 and SMAD3 phosphorylation [15]. Other studies have shown that MSC treatment reduces proinflammatory cytokines such as TNF-α and IL-6 while increasing anti-inflammatory cytokines like IL-10 [32]. In DMD muscle, ongoing infiltration by M1 macrophages and activated T cells creates a cycle of necrosis, inflammation, and fibrosis [33]. IL-10 secreted by ASCs induces anti-inflammatory genes such as suppressor of cytokine signaling 3 (SOCS3) and Arginase 1 (Arg-1), inhibits nuclear factor κB (NF-κB) signaling, and promotes macrophage polarization toward the M2 phenotype, reducing TNF-α and IL-1β production.　Moreover, several factors secreted by ASCs have been reported to exert therapeutic effects in muscular dystrophy. For instance, VEGF-A promotes vascular density maintenance and activates satellite cells [34], while IGF-1 facilitates muscle hypertrophy and protein synthesis [35].

In this study, the continuous administration group showed the lowest levels of TGF-β1, IL-6, IL-1β, and TNF-α, whereas IL-10, VEGF-A, and IGF-1 levels were highest. These findings are consistent with previous studies and suggest that anti-inflammatory factors secreted by ASCs contributed to the therapeutic effects observed. The results also indicate that continuous administration from an early stage may enhance efficacy. Furthermore, qPCR analysis of mRNA extracted from ASCs confirmed the expression of these anti-inflammatory factors, supporting the role of ASC-derived secretory factors in mediating these effects.

Building upon these anti-inflammatory and regenerative pathways, we further explored how ASC treatment might intersect with mechanisms of cellular senescence. In DMD, the breakdown of muscle cells leads to the leakage of enzymes, triggering a natural immune response [36]. The persistent infiltration of inflammatory cells also increases inflammatory cytokines, resulting in cellular senescence due to oxidative stress [37, 38]. In DMD, senescence in mesenchymal progenitor cells results in the secretion of SASP, inhibiting satellite cell pool maintenance and myogenic differentiation programs [25]. Although previous studies have shown the therapeutic effects of MSCs or MSC-derived exosomes in various models, such as renal infarction model rats [39], mouse renal tubular epithelial cells subjected to radiation-induced senescence [40], and osteoarthritis model rats [41], studies linking stem cell therapy for DMD with SASP are lacking. In this study, although no statistically significant differences were observed, MSC administration appeared to reduce SASP levels in DMD model rats, potentially alleviating the phenotype. It is possible that MSCs suppressed the inflammatory response and oxidative stress associated with cellular senescence in DMD, as well as mitigated the inhibition of satellite cell pool maintenance and myogenic differentiation programs caused by cellular senescence. To further analyze the underlying mechanisms, we assessed the expression levels of CDKN2A, which encodes p16 and p19, and CDKN1A, which encodes p21. Increased expression of the former leads to G1 phase cell cycle arrest, accumulation of DNA damage, reduced free radical scavenging capacity, and senescence of progenitor stem cells [42, 43]. This pattern indicates irreversible cellular senescence. In contrast, the latter is transcriptionally activated by p53 and also induces G1 arrest, allowing time for DNA repair. Additionally, during the differentiation of myoblasts into satellite cells, CDKN1A promotes cell cycle exit by inhibiting cyclin-dependent kinase activity, thus facilitating terminal differentiation of muscle cells. This function differs from CDKN2A in that it is associated not only with cellular senescence but also with the promotion of differentiation [44]. In this study, the continuous group exhibited low CDKN2A and high CDKN1A expression. This result suggests that the treatment simultaneously suppressed irreversible senescence and enhanced myoblast differentiation, providing a two-tiered regulatory effect and offering molecular and cellular biological evidence for the therapeutic mechanism. In contrast, the early and late groups showed high CDKN2A and low CDKN1A expression levels. These patterns likely reflect limited therapeutic efficacy of MSCs, with accumulation of senescent cells and insufficient regenerative potential. These findings are consistent with the SASP analysis results and suggest that expression of these genes may serve as molecular targets for assessing therapeutic responsiveness in MSC treatment. Our correlation analysis corroborates two molecular circuits that have been documented in diverse fibrotic and regenerative settings. The first circuit is a fibro‑inflammatory loop that couples TGF‑β1, IL‑1β, CTGF, MMP‑2 and TNF‑α. TGF‑β1 is recognized as a master driver of fibrosis and myofibroblast differentiation [45]. CTGF is a direct downstream effector of TGF‑β1 and amplifies its fibrogenic actions; genetic or pharmacological blockade of CTGF attenuates TGF‑β‑induced scarring in vivo [46]. IL‑1β and TNF‑α released from activated immune cells promote CTGF and TGF‑β1 expression in fibroblasts, thereby establishing a positive feedback loop that sustains inflammation and matrix production [45]. In our data set these five genes clustered tightly and correlated negatively with grip strength, indicating that continuous ASC treatment attenuates the entire fibro‑inflammatory cascade rather than isolated components. The second circuit is a regenerative/anti‑inflammatory module centered on CDKN1A (p21) together with VEGF‑A, IGF‑1 and IL‑10. p21 deletion in adult mice permits scar‑free regeneration of skin, cartilage [47]. At the immune level, p21 drives the switch of macrophages toward an M2‑like, IL‑10‑high phenotype via p50-p50 NF-κB pathways [48]. IGF‑1 reinforces this environment by increasing IL‑10 expression and promoting M2 polarization, thereby accelerating muscle repair [49]. IL‑10 itself can induce p53/p21‑dependent senescence in activated stellate cells, limiting collagen deposition and favoring resolution of liver fibrosis [50]. The strong positive correlations we observed among p21, VEGF‑A, IGF‑1 and IL‑10—most pronounced in the continuous‑dose group—fit this model and suggest that ASC therapy activates a p21‑centered repair‑promoting pathway. Several studies also show that mesenchymal stem cells directly promote macrophage polarization toward the M2 phenotype through paracrine mediators such as PGE₂ and TSG‑6 [51]. This provides an additional mechanism by which continuous ASC administration may enhance the regenerative microenvironment observed in this study.

Together, the opposing molecular shifts—decreased activity of the TGF‑β1/IL‑1β/CTGF/MMP‑2/TNF‑α loop and increased activity of the p21/VEGF‑A/IGF‑1/IL‑10 loop—offer a molecular basis for the superior functional and histological outcomes of continuous ASC dosing. The composite signature of elevated p21, VEGF‑A, IGF‑1 and IL‑10 together with reduced TGF‑β1 therefore may serve as a promising biomarker panel for effective MSC therapy.

The therapeutic effects observed—reduced CK levels, preserved endurance, and suppression of fibrosis, necrosis, and inflammation—are consistent with previous findings. At the molecular and cellular level, the concordant shifts in senescence markers and in cytokines substantiate these functional benefits and clarify the underlying biology. This study also highlights aspects that have not been well characterized in earlier research. Early-only administration was associated with negative effects, and the findings emphasize the importance of treatment timing. These results may help inform the design of future therapeutic strategies.

Taken together, these findings provide novel insights into the temporal dynamics and molecular underpinnings of stem cell therapy in DMD, reinforcing the therapeutic potential of continuous ASC administration. However, despite these encouraging outcomes, several limitations should be noted. The study concentrated on administration timing and delivery routes and did not include comparisons to standard treatments such as corticosteroids. It also lacked in-depth analysis of molecular and cellular mechanisms. In neonatal transplantation, MSCs had to be administered prior to genotyping, and only after administration could the genotype be confirmed. Since only 25% of the offspring were DMD males, this substantially limited the number of subjects and made it difficult to increase the sample size. As a result, the transplantation groups remained small. While we acknowledge that a larger sample size is generally desirable, we respectfully note that our current study design still provides sufficient statistical power to support our conclusions. Specifically, post-hoc power analyses demonstrated that key outcomes had adequate effect sizes and power values. These values indicate that even with *n* = 5, the analyses were sufficiently powered to detect meaningful differences among groups. Furthermore, the findings across functional, histological, and molecular endpoints were internally consistent and biologically coherent. Therefore, we believe that the sample size used in this study was acceptable and did not compromise the scientific validity of our conclusions. The study was further limited by the absence of long-term follow-up beyond day 56. However, postnatal day 56 in DMD rats corresponds to an advanced stage of pathology, with pronounced muscle necrosis and fibrosis. Compared to the mdx mouse, the DMD rat model exhibits more rapid disease progression [17]. In several peer reviewed studies, outcome assessments between four and twelve weeks, including around day 56, are included as part of broader longitudinal designs [52–54]. Based on these pathological features and literature precedents, we judged day 56 to be a scientifically appropriate endpoint for assessing treatment efficacy. Nevertheless, future studies should incorporate extended observation periods to assess effects on chronic fibrosis and cardiac involvement.

## Conclusion

This study is the first to vary the transplantation timing to search for the optimal treatment method and to perform stem cell transplantation, including the use of a 1-day-old donor. Early detection and treatment of DMD can suppress disease progression; however, early administration followed by discontinuation of treatment might result in a worse prognosis compared to no treatment. Furthermore, cellular senescence associated factors and histological analyses suggest that MSC administration suppresses cellular senescence, fibrosis, necrosis, and regeneration in DMD. The parallel molecular signature (low CDKN2A together with high CDKN1A, VEGF-A, IGF-1 and IL-10) corroborates these tissue-level benefits and clarifies the molecular and cellular mechanisms that underlie the therapeutic effects observed. These insights provide a foundation for optimizing therapeutic strategies for DMD and support the critical role of paracrine and endocrine effects over direct cellular engraftment in achieving these outcomes.

## Supplementary Information

Below is the link to the electronic supplementary material.

Supplementary Material 1

## Data Availability

All data generated or analyzed during this study are included in this published article and its supplementary information files. No sequencing data were generated in this study.

## Abbreviations

ASC: Adipose-derived stem cell
CDKN: Cyclin-dependent kinase inhibitor
CK: Creatine kinase
CTGF: Connective tissue growth factor
DMD: Duchenne muscular dystrophy
eGFP: Enhanced green fluorescent protein
eMHC: Embryonic myosin heavy chain
FBS: Fetal bovine serum
GAPDH: Glyceraldehyde-3-phosphate dehydrogenase
GFP: Green fluorescent protein
IGF-1: Insulin-like growth factor 1
IL: Interleukin
MMP-2: Matrix metalloproteinase-2
MSCs: Mesenchymal stem cells
PBS: Phosphate-buffered saline
SASP: Senescence-associated secretory phenotype
TA: Tibialis anterior
TGF-β1: Transforming growth factor beta 1
TNF-α: Tumor necrosis factor alpha
VEGF-A: Vascular endothelial growth factor A

## References

[1] Duan D, Goemans N, Takeda S, Mercuri E, Aartsma-Rus A. Duchenne muscular dystrophy. Nat Rev Dis Primers. 2021;7:13. 33602943 10.1038/s41572-021-00248-3 PMC10557455

[2] Roberts TC, Wood MJA, Davies KE. Therapeutic approaches for Duchenne muscular dystrophy. Nat Rev Drug Discov. 2023;22:917–34. 37652974 10.1038/s41573-023-00775-6

[3] Griggs RC, Herr BE, Reha A, Elfring G, Atkinson L, Cwik V, et al. Corticosteroids in Duchenne muscular dystrophy: major variations in practice. Muscle Nerve. 2013;48:27–31. 23483575 10.1002/mus.23831

[4] Flanigan KM, Vetter TA, Simmons TR, Iammarino M, Frair EC, Rinaldi F, et al. A first-in-human phase i/iia gene transfer clinical trial for Duchenne muscular dystrophy using rAAVrh74.MCK.GALGT2. Mol Ther Methods Clin Dev. 2022;27:47–60. 36186954 10.1016/j.omtm.2022.08.009 PMC9483573

[5] Clemens PR, Rao VK, Connolly AM, Harper AD, Mah JK, McDonald CM, et al. Long-term functional efficacy and safety of viltolarsen in patients with Duchenne muscular dystrophy. J Neuromuscul Dis. 2022;9:493–501. 35634851 10.3233/JND-220811 PMC9398057

[6] Elangkovan N, Dickson G. Gene therapy for Duchenne muscular dystrophy. J Neuromuscul Dis. 2021;8:S303–16. 34511510 10.3233/JND-210678 PMC8673537

[7] Matthews E, Brassington R, Kuntzer T, Jichi F, Manzur AY. Corticosteroids for the treatment of Duchenne muscular dystrophy. Cochrane Database Syst Rev. 2016;2016:Cd003725. 27149418 10.1002/14651858.CD003725.pub4 PMC8580515

[8] Feng SW, Lu XL, Liu ZS, Zhang YN, Liu TY, Li JL, et al. Dynamic distribution of bone marrow-derived mesenchymal stromal cells and change of pathology after infusing into Mdx mice. Cytotherapy. 2008;10:254–64. 18418771 10.1080/14653240802020381

[9] Siemionow M, Brodowska S, Langa P, Zalants K, Kozlowska K, Grau-Kazmierczak W, et al. Long-term biodistribution and safety of human dystrophin expressing chimeric cell therapy after systemic-intraosseous administration to Duchenne muscular dystrophy model. Arch Immunol Ther Exp (Warsz). 2022;70:20. 35978142 10.1007/s00005-022-00656-7 PMC9385806

[10] Sitzia C, Farini A, Jardim L, Razini P, Belicchi M, Cassinelli L, et al. Adaptive immune response impairs the efficacy of autologous transplantation of engineered stem cells in dystrophic dogs. Mol Ther. 2016;24:1949–64. 27506452 10.1038/mt.2016.163 PMC5154479

[11] Zhang Y, Zhu Y, Li Y, Cao J, Zhang H, Chen M, et al. Long-term engraftment of myogenic progenitors from adipose-derived stem cells and muscle regeneration in dystrophic mice. Hum Mol Genet. 2015;24:6029–40. 26264578 10.1093/hmg/ddv316

[12] McDonald CM, Marbán E, Hendrix S, Hogan N, Ruckdeschel Smith R, Eagle M, et al. Repeated intravenous cardiosphere-derived cell therapy in late-stage Duchenne muscular dystrophy (HOPE-2): a multicentre, randomised, double-blind, placebo-controlled, phase 2 trial. Lancet. 2022;399:1049–58. 35279258 10.1016/S0140-6736(22)00012-5

[13] Dai A, Baspinar O, Yeşilyurt A, Sun E, Aydemir Ç, Öztel ON, et al. Efficacy of stem cell therapy in ambulatory and nonambulatory children with Duchenne muscular dystrophy - Phase I-II. Degener Neurol Neuromuscul Dis. 2018;8:63–77. 30498389 10.2147/DNND.S170087 PMC6207384

[14] Heydemann A, Bieganski G, Wachowiak J, Czarnota J, Niezgoda A, Siemionow K, et al. Dystrophin expressing chimeric (DEC) cell therapy for Duchenne muscular dystrophy: a first-in-human study with minimum 6 months follow-up. Stem Cell Rev Rep. 2023;19:1340–59. 37000376 10.1007/s12015-023-10530-4 PMC10366026

[15] Park SE, Jeong JB, Oh SJ, Kim SJ, Kim H, Choi A, et al. Wharton’s jelly-derived mesenchymal stem cells reduce fibrosis in a mouse model of Duchenne muscular dystrophy by upregulating MicroRNA 499. Biomedicines. 2021;9:1089. 34572277 10.3390/biomedicines9091089 PMC8469349

[16] Arakawa A, Ichikawa H, Kubo T, Motoi N, Kumamoto T, Nakajima M, et al. Vaginal transmission of cancer from mothers with cervical cancer to infants. N Engl J Med. 2021;384:42–50. 33406329 10.1056/NEJMoa2030391

[17] Nakamura K, Fujii W, Tsuboi M, Tanihata J, Teramoto N, Takeuchi S, et al. Generation of muscular dystrophy model rats with a crispr/cas system. Sci Rep. 2014;4:5635. 25005781 10.1038/srep05635 PMC4088098

[18] Ryu B, Sekine H, Homma J, Kobayashi T, Kobayashi E, Kawamata T, et al. Allogeneic adipose-derived mesenchymal stem cell sheet that produces neurological improvement with angiogenesis and neurogenesis in a rat stroke model. J Neurosurg. 2019;132:442–55. 30797215 10.3171/2018.11.JNS182331

[19] Mohamed-Ahmed S, Yassin MA, Rashad A, Espedal H, Idris SB, Finne-Wistrand A, et al. Comparison of bone regenerative capacity of donor-matched human adipose-derived and bone marrow mesenchymal stem cells. Cell Tissue Res. 2021;383:1061–75. 33242173 10.1007/s00441-020-03315-5 PMC7960590

[20] Brooks AES, Iminitoff M, Williams E, Damani T, Jackson-Patel V, Fan V, et al. Ex vivo human adipose tissue derived mesenchymal stromal cells (ASC) are a heterogeneous population that demonstrate rapid culture-induced changes. Front Pharmacol. 2019;10:1695. 32153389 10.3389/fphar.2019.01695 PMC7044177

[21] Kihara Y, Homma J, Takagi R, Ishigaki K, Nagata S, Yamato M. Laminin-221-derived recombinant fragment facilitates isolation of cultured skeletal myoblasts. Regenerative Therapy. 2022;20:147–56. 35620637 10.1016/j.reth.2022.04.006 PMC9111930

[22] Agarwal M, Sharma A, Kumar P, Kumar A, Bharadwaj A, Saini M et al. Myosin heavy chain-embryonic regulates skeletal muscle differentiation during mammalian development. Development. 2020;147. 10.1242/dev.184507 PMC7157585 32094117

[23] Kuilman T, Michaloglou C, Vredeveld LC, Douma S, van Doorn R, Desmet CJ, et al. Oncogene-induced senescence relayed by an interleukin-dependent inflammatory network. Cell. 2008;133:1019–31. 18555778 10.1016/j.cell.2008.03.039

[24] Tominaga K, Suzuki HI. TGF-β signaling in cellular senescence and aging-related pathology. Int J Mol Sci. 2019;20. 10.3390/ijms20205002 PMC6834140 31658594

[25] Sugihara H, Teramoto N, Nakamura K, Shiga T, Shirakawa T, Matsuo M, et al. Cellular senescence-mediated exacerbation of Duchenne muscular dystrophy. Sci Rep. 2020;10:16385. 33046751 10.1038/s41598-020-73315-6 PMC7550355

[26] Rodriguez AM, Pisani D, Dechesne CA, Turc-Carel C, Kurzenne JY, Wdziekonski B, et al. Transplantation of a multipotent cell population from human adipose tissue induces dystrophin expression in the immunocompetent Mdx mouse. J Exp Med. 2005;201:1397–405. 15867092 10.1084/jem.20042224 PMC2213197

[27] Vieira NM, Valadares M, Zucconi E, Secco M, Bueno CR Jr., Brandalise V, et al. Human adipose-derived mesenchymal stromal cells injected systemically into GRMD dogs without immunosuppression are able to reach the host muscle and express human dystrophin. Cell Transpl. 2012;21:1407–17. 10.3727/096368911X 23168016

[28] Kerkis I, Ambrosio CE, Kerkis A, Martins DS, Zucconi E, Fonseca SA, et al. Early transplantation of human immature dental pulp stem cells from baby teeth to golden retriever muscular dystrophy (GRMD) dogs: local or systemic? J Transl Med. 2008;6:35. 18598348 10.1186/1479-5876-6-35 PMC2529267

[29] Ullah M, Liu DD, Thakor AS. Mesenchymal stromal cell homing: mechanisms and strategies for improvement. iScience. 2019;15:421–38. 31121468 10.1016/j.isci.2019.05.004 PMC6529790

[30] Rodríguez-Cruz M, Almeida-Becerril T, Atilano-Miguel S, Cárdenas-Conejo A, Bernabe-García M. Natural history of serum enzyme levels in Duchenne muscular dystrophy and implications for clinical practice. Am J Phys Med Rehabil. 2020;99:1121–8. 32520799 10.1097/PHM.0000000000001500

[31] Moghadam-Kia S, Oddis CV, Aggarwal R. Approach to asymptomatic creatine kinase elevation. Cleve Clin J Med. 2016;83:37–42. 26760521 10.3949/ccjm.83a.14120 PMC4871266

[32] Torres-Torrillas M, Rubio M, Damia E, Cuervo B, Del Romero A, Peláez P et al. Adipose-derived mesenchymal stem cells: a promising tool in the treatment of musculoskeletal diseases. Int J Mol Sci. 2019;20. 10.3390/ijms20123105 PMC6627452 31242644

[33] Nitahara-Kasahara Y, Nakayama S, Kimura K, Yamaguchi S, Kakiuchi Y, Nito C, et al. Immunomodulatory amnion-derived mesenchymal stromal cells preserve muscle function in a mouse model of Duchenne muscular dystrophy. Stem Cell Res Ther. 2023;14:108. 37106393 10.1186/s13287-023-03337-0 PMC10142496

[34] Baumann M, Gumpold C, Mueller-Felber W, Schoser B, Haberler C, Loescher WN, et al. Pattern of myogenesis and vascular repair in early and advanced lesions of juvenile dermatomyositis. Neuromuscul Disord. 2018;28:973–85. 30389421 10.1016/j.nmd.2018.09.002

[35] Barton ER, Morris L, Musaro A, Rosenthal N, Sweeney HL. Muscle-specific expression of insulin-like growth factor I counters muscle decline in Mdx mice. J Cell Biol. 2002;157:137–48. 11927606 10.1083/jcb.200108071 PMC2173262

[36] Rosenberg AS, Puig M, Nagaraju K, Hoffman EP, Villalta SA, Rao VA, et al. Immune-mediated pathology in Duchenne muscular dystrophy. Sci Transl Med. 2015;7:299rv4. 10.1126/scitranslmed.aaa7322 PMC5951380 26246170

[37] Braumüller H, Wieder T, Brenner E, Aßmann S, Hahn M, Alkhaled M, et al. T-helper-1-cell cytokines drive cancer into senescence. Nature. 2013;494:361–5. 23376950 10.1038/nature11824

[38] Reimann M, Lee S, Loddenkemper C, Dörr JR, Tabor V, Aichele P, et al. Tumor stroma-derived TGF-beta limits myc-driven lymphomagenesis via Suv39h1-dependent senescence. Cancer Cell. 2010;17:262–72. 20227040 10.1016/j.ccr.2009.12.043

[39] Rodrigues CE, Capcha JM, de Bragança AC, Sanches TR, Gouveia PQ, de Oliveira PA, et al. Human umbilical cord-derived mesenchymal stromal cells protect against premature renal senescence resulting from oxidative stress in rats with acute kidney injury. Stem Cell Res Ther. 2017;8:19. 28129785 10.1186/s13287-017-0475-8 PMC5273809

[40] Liao CM, Luo T, von der Ohe J, de Juan Mora B, Schmitt R, Hass R. Human MSC-derived exosomes reduce cellular senescence in renal epithelial cells. Int J Mol Sci. 2021;22. 10.3390/ijms222413562 PMC8709122 34948355

[41] Lee EM, Kim AY, Lee EJ, Park JK, Lee MM, Hwang M, et al. Therapeutic effects of mouse adipose-derived stem cells and losartan in the skeletal muscle of injured Mdx mice. Cell Transpl. 2015;24:939–53. 10.3727/096368914X678599 24593934

[42] Rayess H, Wang MB, Srivatsan ES. Cellular senescence and tumor suppressor gene p16. Int J Cancer. 2012;130:1715–25. 22025288 10.1002/ijc.27316 PMC3288293

[43] Gan Q, Huang J, Zhou R, Niu J, Zhu X, Wang J, et al. PPAR{gamma} accelerates cellular senescence by inducing p16INK4{alpha} expression in human diploid fibroblasts. J Cell Sci. 2008;121:2235–45. 18544633 10.1242/jcs.026633

[44] Guo K, Wang J, Andrés V, Smith RC, Walsh K. MyoD-induced expression of p21 inhibits cyclin-dependent kinase activity upon myocyte terminal differentiation. Mol Cell Biol. 1995;15:3823–9. 7791789 10.1128/mcb.15.7.3823 PMC230621

[45] Deng Z, Fan T, Xiao C, Tian H, Zheng Y, Li C, et al. TGF-β signaling in health, disease and therapeutics. Signal Transduct Target Therapy. 2024;9:61. 10.1038/s41392-024-01764-w PMC10958066 38514615

[46] Ren M, Yao S, Chen T, Luo H, Tao X, Jiang H et al. Connective tissue growth factor: regulation, diseases, and drug discovery. Int J Mol Sci. 2024;25. 10.3390/ijms25094692 PMC11083620 38731911

[47] Bedelbaeva K, Snyder A, Gourevitch D, Clark L, Zhang XM, Leferovich J, et al. Lack of p21 expression links cell cycle control and appendage regeneration in mice. Proc Natl Acad Sci U S A. 2010;107:5845–50. 20231440 10.1073/pnas.1000830107 PMC2851923

[48] Rackov G, Hernández-Jiménez E, Shokri R, Carmona-Rodríguez L, Mañes S, Álvarez-Mon M, et al. p21 mediates macrophage reprogramming through regulation of p50-p50 NF-κB and IFN-β. J Clin Invest. 2016;126:3089–103. 27427981 10.1172/JCI83404 PMC4966310

[49] Tonkin J, Temmerman L, Sampson RD, Gallego-Colon E, Barberi L, Bilbao D, et al. Monocyte/Macrophage-derived IGF-1 orchestrates murine skeletal muscle regeneration and modulates autocrine polarization. Mol Ther. 2015;23:1189–200. 25896247 10.1038/mt.2015.66 PMC4817788

[50] Strle K, McCusker RH, Johnson RW, Zunich SM, Dantzer R, Kelley KW. Prototypical anti-inflammatory cytokine IL-10 prevents loss of IGF-I-induced myogenin protein expression caused by IL-1beta. Am J Physiol Endocrinol Metab. 2008;294:E709–18. 18270299 10.1152/ajpendo.00662.2007 PMC2951888

[51] Song W-J, Li Q, Ryu M-O, Ahn J-O, Ha Bhang D, Chan Jung Y, et al. TSG-6 secreted by human adipose tissue-derived mesenchymal stem cells ameliorates DSS-induced colitis by inducing M2 macrophage polarization in mice. Sci Rep. 2017;7:5187. 28701721 10.1038/s41598-017-04766-7 PMC5507867

[52] Ouisse L-H, Remy S, Lafoux A, Larcher T, Tesson L, Chenouard V, et al. Immunophenotype of a rat model of duchenne’s disease and demonstration of improved muscle strength after anti-CD45RC antibody treatment. Front Immunol. 2019;10:2019. 31552055 10.3389/fimmu.2019.02131 PMC6746111

[53] Sato M, Goto M, Yamanouchi K, Sakurai H. A new immunodeficient Duchenne muscular dystrophy rat model to evaluate engraftment after human cell transplantation. Front Physiol. 2023;14:1094359. 37101699 10.3389/fphys.2023.1094359 PMC10123282

[54] Baine S, Wier C, Lemmerman L, Cooper-Olson G, Kempton A, Haile A, et al. Long-term survival and myocardial function following systemic delivery of delandistrogene moxeparvovec in DMD(MDX) rats. Hum Gene Ther. 2024;35:978–88. 39607794 10.1089/hum.2024.013 PMC11659437

