# Supplementary material for: Continuous adipose-derived stem cell therapy from the neonatal stage effectively reduces Duchenne muscular dystrophy symptoms in rats
Source: Stem Cell Res Ther. 2025 Aug 26;16:452. doi: 10.1186/s13287-025-04594-x (PMC12382225; doi:10.1186/s13287-025-04594-x)
Supplement: Supplementary file 1 — Supplementary Material 1 [file 13287_2025_4594_MOESM1_ESM.pdf]

Supplementary Figure 1

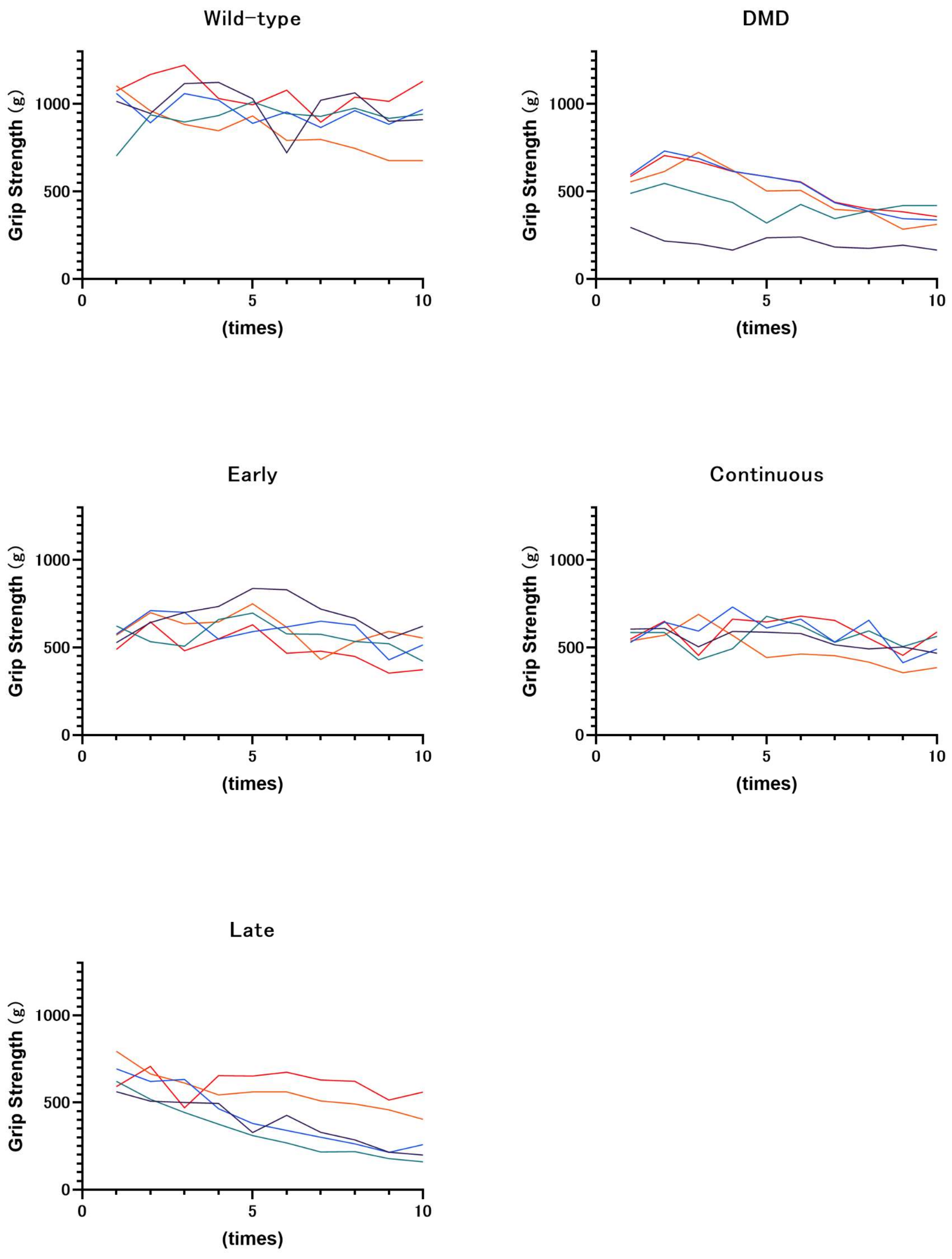

Supplementary Figure 1: Grip Strength Trend
This graph illustrates the grip strength measurements for all groups, showing a consistent decline in grip strength over time in the Duchenne Muscular Dystrophy control and late administration groups. Meanwhile, the remaining groups generally maintained stable grip strength throughout the study, with minor variations.

Supplementary Figure 2

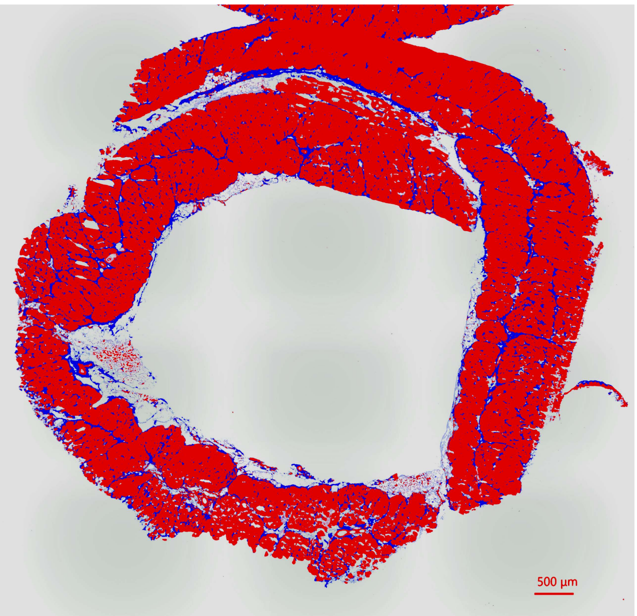

a) Early Administration group

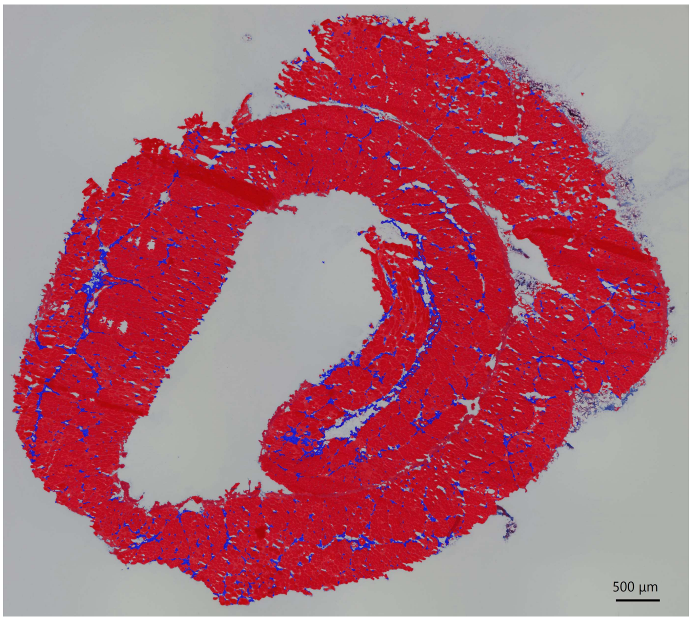

b) Continuous Administration group

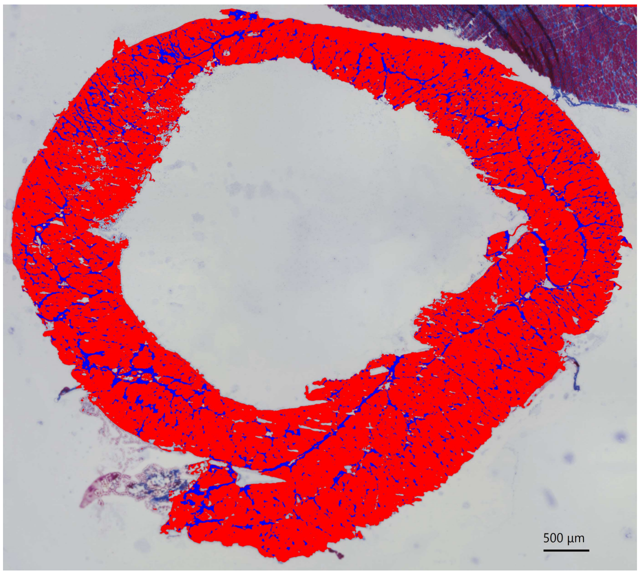

c) Late Administration group

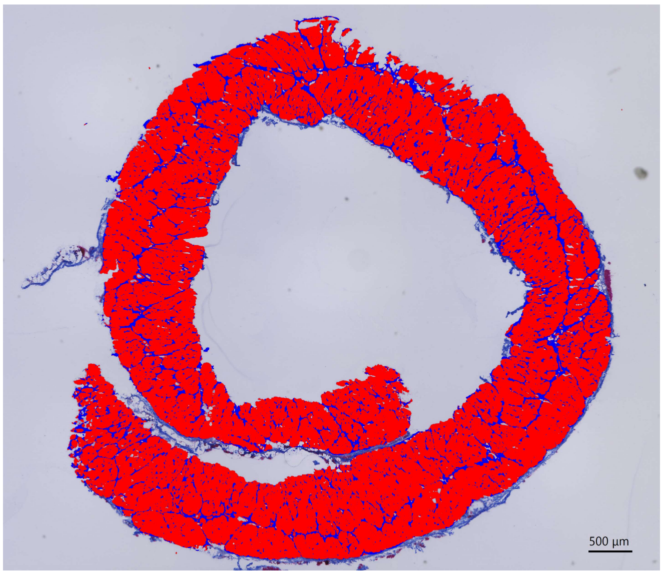

d) DMD Control group

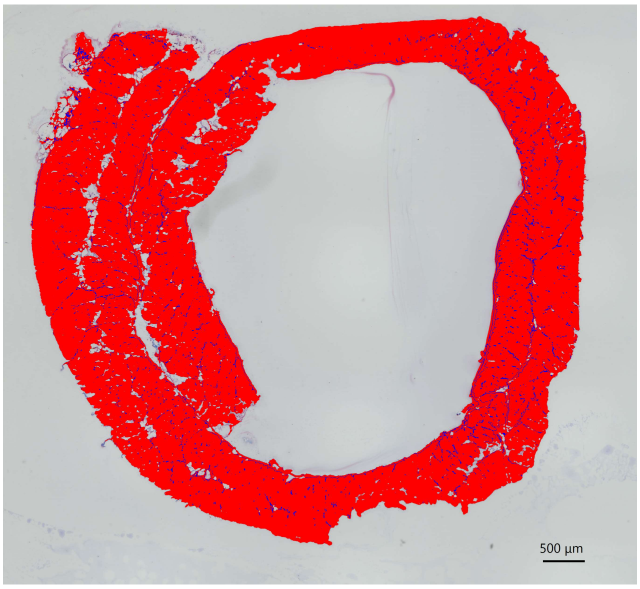

e) Wild-type Control group

Supplementary Figure 2: Color-Segmented Masson's Trichrome Staining
The blue areas and other regions in the Masson’s Trichrome-stained sections were segmented by color using image analysis software. The ratio of the blue area to the total area was calculated to assess the degree of fibrosis.

Supplementary Figure 3

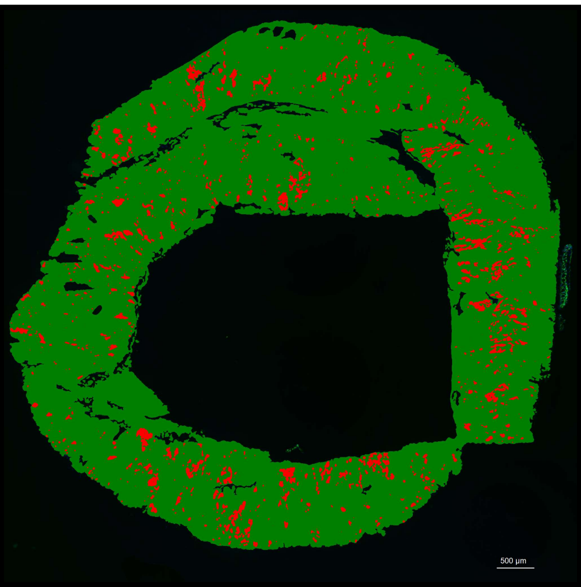

a) Early Administration group

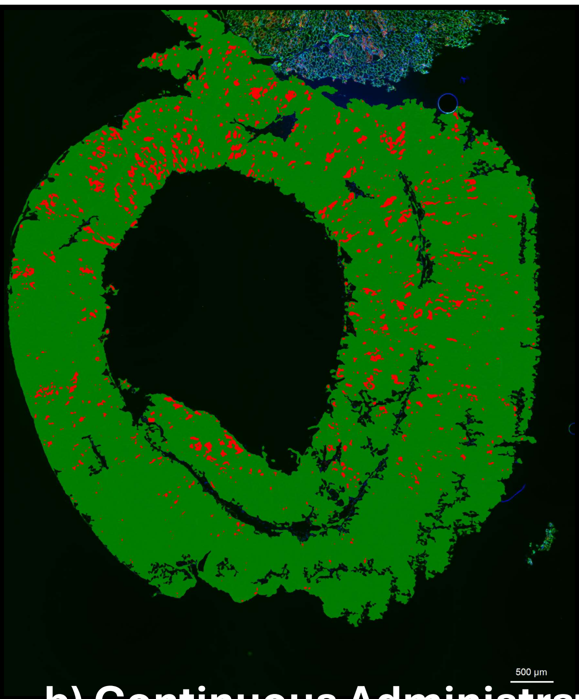

b) Continuous Administration group

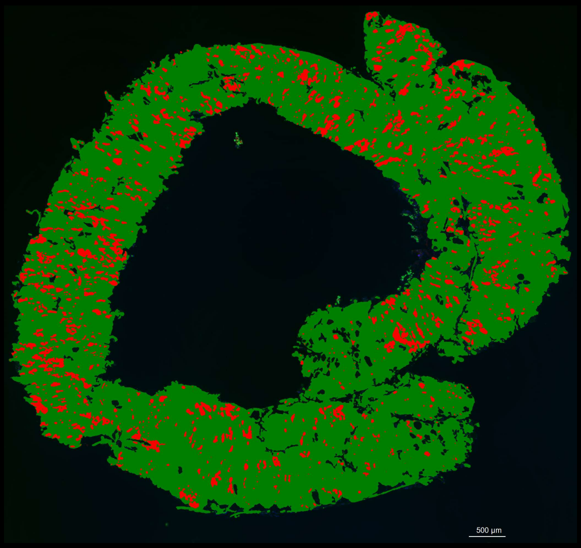

c) Late Administration group

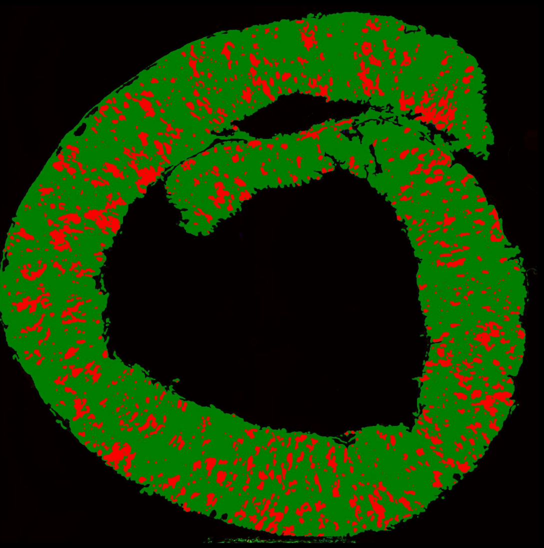

d) DMD Control group

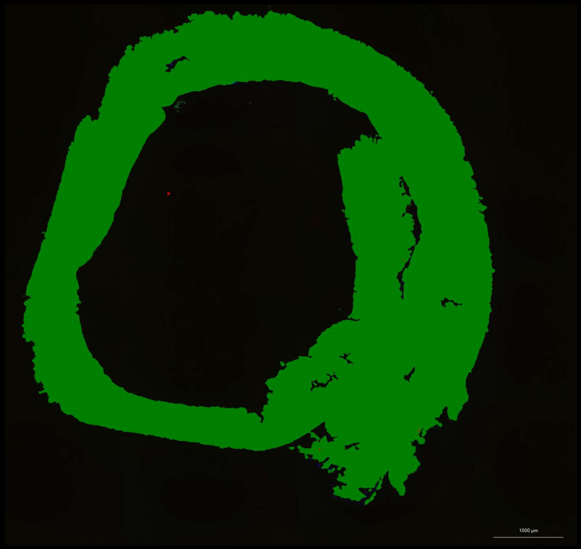

e) Wild-type Control group

Supplementary Figure 3: Color segmented Embryonic Myosin Heavy Chain-Positive Fibers

The fibers were stained with an anti-embryonic myosin heavy chain (eMHC) antibody, and positive (regenerative) and non-positive fibers were segmented by color using image analysis software. The ratio of the area occupied by red-stained positive fibers to the total area was calculated to assess the extent of necrosis and regeneration.

Supplementary Figure 4
ISH for EGFP mRNA detection

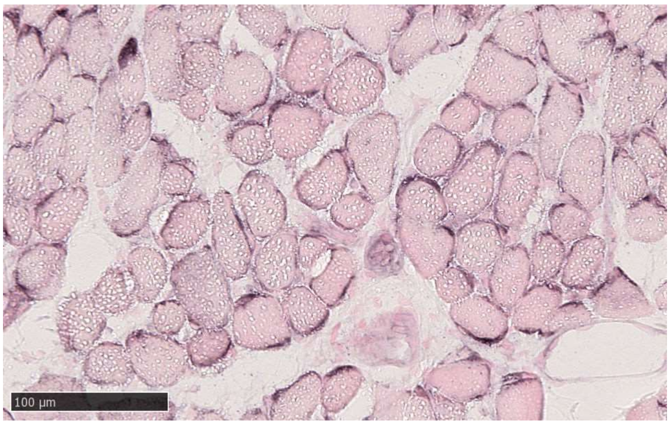

a) GFP Rat (Positive Control)

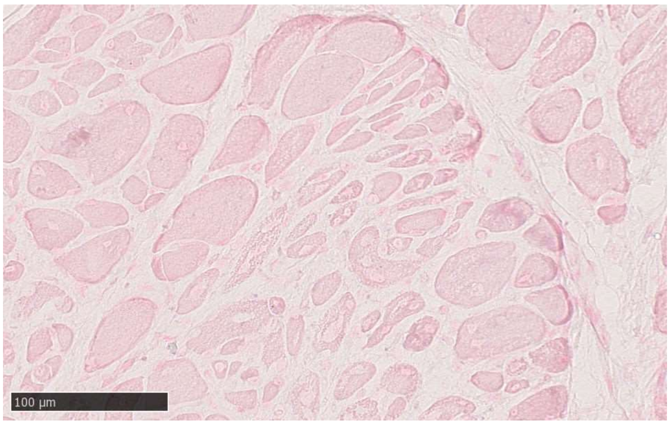

b) Continuous Group

a) The areas stained black indicate EGFP-specific staining. b) In the continuous administration group, no muscle fibers showing EGFP-specific staining were observed.

EGFP probe used for ISH:
GTGAACCGCATCGAGCTGAAGGGCATCGACTTCAAGGAGGACGGCAAC
ATCCTGGGGCACAAGCTGGAGTACAACTACAACAGCCACAACGTCTATA
TCATGGCCGACAAGCAGAAGAACGGCATCAAGGTGAACTTCAAGATCC
GCCACAACATCGAGGACGGCAGCGTGCAGCTCGCCGACCACTACCAGC
AGAACACCCCCATCGGCGACGGCCCCGTGCTGCTGCCCGACAACCACTA
CCTGAGCACCCAGTCCGCCCTGAGCAAAGACCCCAACGAGAAGCGCGA
TCACATGGTCCTGCTGGAGTTCGTGACCGCCGCCGGGATCACTCTCGGC
ATGGACGAGCTGTACAAGTAA

Supplementary Table 1
Antibodies and isotype controls used for flow cytometric analysis.

Name	Source	Cat. No.
Aanti-CD34 Monoclonal Antibody	Invitrogen	11-0341-82
Anti-mouse/rat CD29 Antibody	BioLegend	102205
Anti-rat CD90/mouse CD90.1 (Thy-1.1) Antibody	BioLegend	202503
Anti-rat CD11b/c Antibody	BioLegend	201805
Anti-rat CD45 Antibody	BioLegend	202205
Anti-rat IgG2a kappa Isotype Control Antibody	Invitrogen	11-4321-80
Anti-armenian Hamster IgG Isotype Control Antibody	BioLegend	400905
Anti-Mouse IgG1, κ Isotype Control Antibody	BioLegend	400107
Anti-Mouse IgG2a, κ Isotype Control Antibody	BioLegend	400207

Supplementary Table 2
Antibodies used for immunofluorescence staining.

Name	Source	Cat. No.	Dilution
Anti-Green Fluorescent Protein Antibody	abcam	ab290	1:500
Anti-Dystrophin-1 Antibody	Novocastra	NCL-DYS1	1:20
Anti-Laminin Antibody	Invitrogen	PA1-16730	1:500
Anti-Myosin Heavy Chain 3 Antibody	Santa Cruz Biotechnology	sc-53091	1:50
Goat Anti-Rabbit IgG H&L (Alexa Fluor® 488)	abcam	ab150077	1:400
Goat Anti-Mouse IgG H&L (Alexa Fluor® 568)	abcam	ab175473	1:400

Supplementary Table 3
TaqMan® gene expression assays used in this study.

Gene Name	Source	Assaay ID
TGF-β1	Thermo Fisher	Rn00572010_m1
IL-6	Thermo Fisher	Rn01410330_m1
IL-1β	Thermo Fisher	Rn00580432_m1
CTGF	Thermo Fisher	Rn00573960_g1
MMP-2	Thermo Fisher	Rn01538170_m1
TNF-α	Thermo Fisher	Rn99999017_m1
CDKN1A	Thermo Fisher	Rn00589996_m1
CDKN2A	Thermo Fisher	Rn00580664_m1
EGFP	Thermo Fisher	Mr04097229_mr
IGF-1	Thermo Fisher	Rn00710306_m1
IL-10	Thermo Fisher	Rn01483988_g1
VEGF-A	Thermo Fisher	Rn01511602_m1
Hprt1	Thermo Fisher	Rn01527840_m1
GAPDH	Thermo Fisher	Rn01775763_g1
